# New novel non-MHC genes were identified for cervical cancer with an integrative analysis approach of transcriptome-wide association study

**DOI:** 10.7150/jca.47918

**Published:** 2021-01-01

**Authors:** Haimiao Chen, Ting Wang, Shuiping Huang, Ping Zeng

**Affiliations:** 1 Department of Epidemiology and Biostatistics, School of Public Health, Xuzhou Medical University, Xuzhou, Jiangsu, 221004, China.; 2 Center for Medical Statistics and Data Analysis, School of Public Health, Xuzhou Medical University, Xuzhou, Jiangsu, 221004, China.

**Keywords:** MetaXcan, cervical cancer, transcriptome-wide association study, Gene expression level, associated genes

## Abstract

Although genome-wide association studies (GWAS) have successfully identified multiple genetic variants associated with cervical cancer, the functional role of those variants is not well understood. To bridge such gap, we integrated the largest cervical cancer GWAS (*N* = 9,347) with gene expression measured in six human tissues to perform a multi-tissue transcriptome-wide association study (TWAS). We identified a total of 20 associated genes in the European population, especially four novel non-MHC genes (i.e. *WDR19*, *RP11-384K6.2*, *RP11-384K6.6* and *ITSN1*). Further, we attempted to validate our results in another independent cervical cancer GWAS from the East Asian population (*N* = 3,314) and re-discovered four genes including *WDR19*, *HLA-DOB*, *MICB* and *OR2B8P*. In our subsequent co-expression analysis, we discovered *SLAMF7* and *LTA* were co-expressed in TCGA tumor samples and showed both *WDR19* and *ITSN1* were enriched in “plasma membrane”. Using the protein-protein interaction analysis we observed strong interactions between the proteins produced by genes that are associated with cervical cancer. Overall, our study identified multiple candidate genes, especially four non-MHC genes, which may be causally associated with the risk of cervical cancer. However, further investigations with larger sample size are warranted to validate our findings in diverse populations.

## Introduction

Cervical cancer is a common female carcinoma and is one of the leading causes of cancer death worldwide 1, especially in developing countries where it brings the major health burden due to limited screening programs available 2-4. Cervical cancer has multiple complex etiologies and is caused by the combination of genetic risk factors and various external environmental exposures. For example, epidemiological evidence suggests that human papillomavirus (HPV) is one of the major risk factors of cervical cancer and contributes to almost all cases 5. However, only ~1% of women finally develop cervical neoplasia with HPV infection 6 because of the strong heritable component and host genetic factors 7.

In the past few years several large-scale genome-wide association studies (GWAS) have discovered a lot of single nucleotide polymorphisms (SNPs) associated with the risk of cervical cancer (Table S1) and provide new insight into the genetic architecture of this type of cancer. However, understanding the underlying mechanism of those identified SNPs on cervical cancer remains considerably challenging as some of them are located within intergenic and the complex major histocompatibility complex (MHC) region 8-10. The difficulty is that the causal genes mediating SNP effects on cervical cancer can be rarely ascertainable with GWAS data alone because of complicated linkage disequilibrium (LD) 11. Therefore, the causal genes and pathways are still not well known and additional genomic functional analyses are required to elucidate the biological mechanism between genetic variants and the risk of cervical cancer 12, 13.

One reasonable explanation for this challenge is that GWAS-associated SNPs may be also associated with molecular-level traits (e.g. gene expression; those variants are also thus referred to as expression quantitative trait loci [eQTL]), which at least partially mediate the causal effects of SNPs on cervical cancer and hold the key to disentangle the genetic foundation of genetic susceptibility to cervical cancer and phenotypic variation. However, in large-scale cervical cancer GWASs genome-wide transcriptome profile is frequently unavailable or unmeasured for all individuals due to high cost and/or unavailability of specimen. Consequently, the association between gene expression and cervical cancer cannot be detected directly 14.

**Figure 1:**
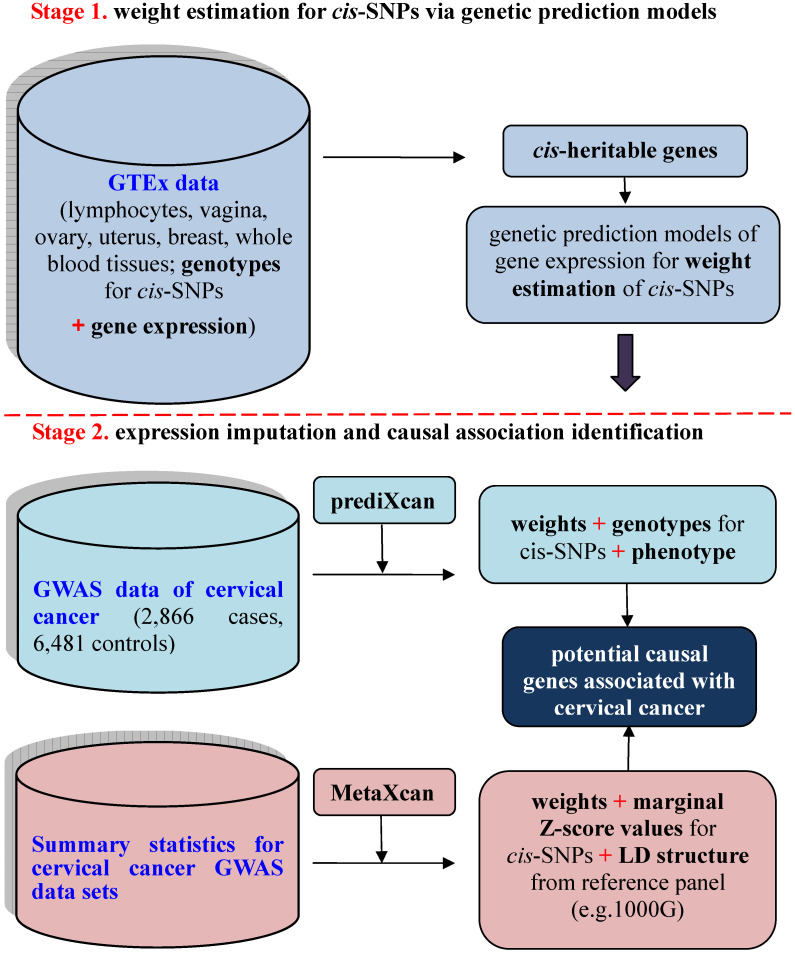
Schematic framework of TWAS using prediXcan with complete data sets or MetaXcan with only summary statistics results. TWAS can be viewed to be a relatively independent two-stage inference procedure: the first stage aims to estimate weights for *cis*-SNPs in an independent transcriptome reference panel using various genetic prediction models with complete data sets of genotypes and gene expressions (i.e. GTEx) (the top panel); the second stage aims to indirectly estimate the causal association between genetically predicted gene expression and a phenotype with weights of *cis*-SNPs obtained from the first stage (the bottom panel).

To investigate the relationship between unmeasured gene expression and cervical cancer in GWAS, we here bridge such a gap by efficiently imputing unmeasured expression based on external transcriptome reference datasets 15, where both gene expression levels and genotypes are available for a relatively small set of individuals. This is the basic idea behind prediXcan 15 — a kind of gene-based association analysis recently developed in the framework of transcriptome-wide association studies (TWAS) 14, 15. Methodologically, prediXcan 15 can be viewed as a relatively independent two-stage inference procedure to prioritize causal genes by integrating transcriptome profile into the association between SNPs and phenotypes. The existence of rich transcriptome datasets from diverse human tissues 16-20 makes it feasible for such an integration. Recently, prediXcan was further extended so that it can be applicable with only summary statistics of GWAS and QTL; the resulting method is referred to as MetaXcan 21. A similar summary-statistics based TWAS method termed FUSION was also proposed meanwhile 14. The schematic framework of TWAS with prediXcan/MetaXcan is shown in Figure 1.

To identify the causal association between genes and cervical cancer, in this study we integrated the transcriptome from multiple GTEx tissues 17, 18 into the cervical cancer GWAS 22 using MetaXcan in the European (EUR) population. A total of 20 genes were identified, among which four novel non-MHC genes might be potentially causal genes associated with the susceptibility of cervical cancer. We also attempted to validate our results in another cervical cancer GWAS from the East Asian (EAS) population 23. Functional analyses were further implemented for those TWAS-identified genes.

## Materials and Methods

### Cervical cancer GWAS datasets and MetaXcan analysis

**Figure 2:**
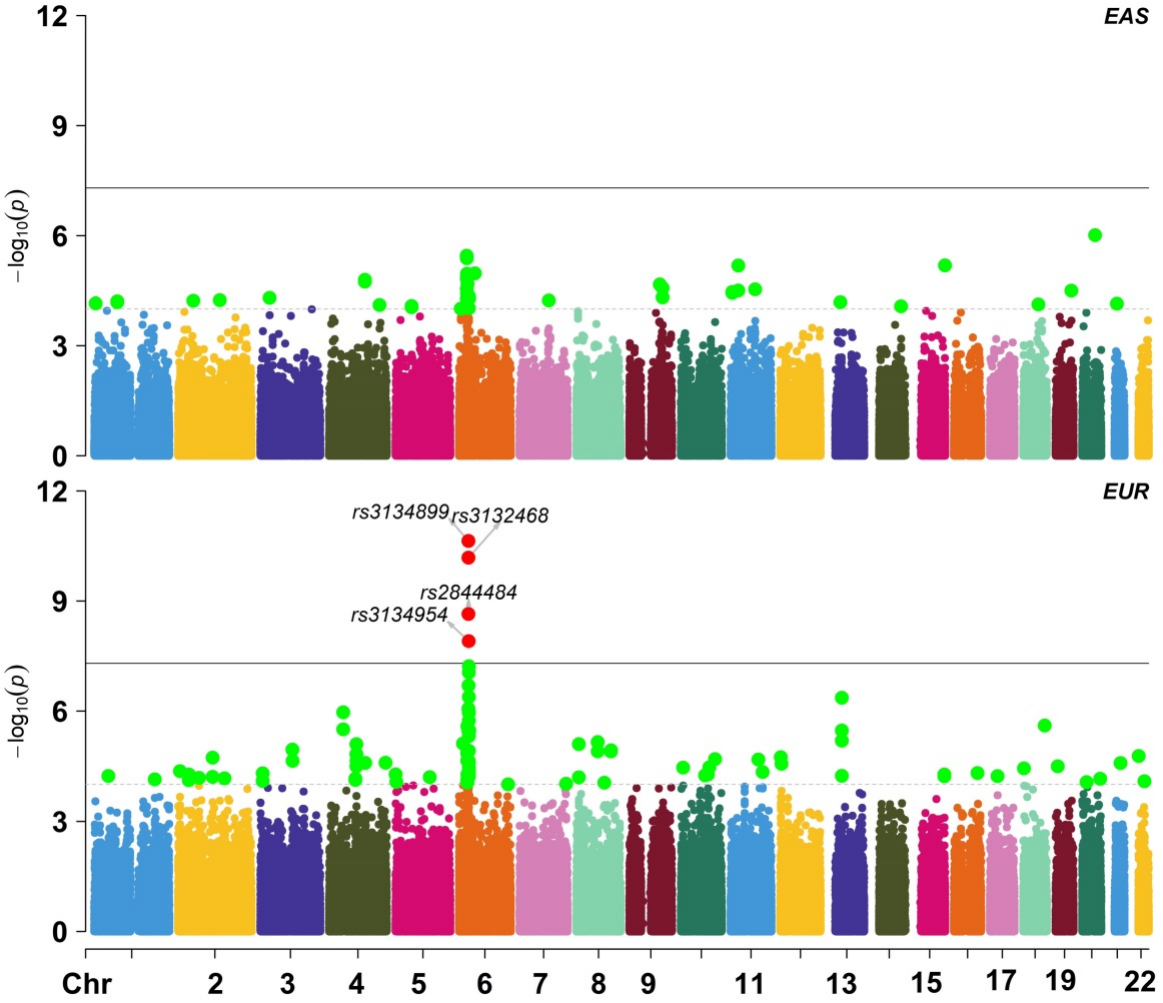
Manhattan plot for East Asian and European ancestry cohorts in the cervical cancer GWAS; EAS: East Asian ancestry; EUR: European ancestry.

We obtained summary statistics of cervical cancer from 22, where a total of 9,347 (2,866 cases and 6,481 controls) individuals of EUR ancestry were included (Figure 2A). The TWAS analysis was implemented with MetaXcan 21 which was in prior trained with genotypes and gene expressions in 48 tissues from the GTEx Project (Table S2) 17, 18. Another reference transcriptome reference panel from Depression Genes and Networks (DGN; 922 whole-blood samples) was also integrated 20. The pre-calculated weights for* cis*-SNPs of each gene can be directly available at http://predictdb.org/ (October, 2019).

Because it has been shown that integrating gene expressions from tissues that are mechanistically unrelated to diseases of interest can lead to spurious associations in TWAS 24, we followed previous work via PubMed retrieval 25 to determinate cervical-cancer related tissues (Table S3). We finally selected gynecological tissues (i.e. vagina, ovary, uterus and breast), whole blood and lymphocytes to be the most relevant tissues for cervical cancer (Supplementary File). In our analysis genes with false discover rate (FDR) < 0.05 were identified to be significant.

As will be shown below, a total of 20 novel genes were detected with MetaXcan in the EUR population. We further validated those genes in a latest cervical cancer GWAS from the EAS population (*N* = 3,314 with 1,320 case and 1,994 control) (Figure 2B) 23. The corresponding transcriptome expression profile came from the tissue of peripheral blood on 98 Japanese individuals 26. The LD matrix was computed with genotypes of 504 EAS individuals in the 1000 Genomes Project 27. The analysis was implemented with a gene-based FUSION-type TWAS approach 14, which is mathematically equivalent to MetaXcan 21.

### Functional analyses for identified genes

Next, combining with previous GWAS-identified genes (Table S1), we implemented three functional analyses for those TWAS-identified genes (Table 1 and Table S4). First, co-expression analysis was performed via weighted gene co-expression network analysis (WGCNA) 28. The visualization of interconnection network was conducted in Cytoscape (http://www.cytoscape.org/) 29 with the topological overlap measurement (TOM) quantifying interconnection among genes 30. Note that, TOM ranges between 0 and 1 indicating interconnection between two genes; greater TOM represents higher interconnection with the same set of genes 30. However, co-expression analysis results from WGCNA may be hard to interpret since copy number variant (CNV) itself is a common feature in cancer 31. In order to adjust for the variation in gene expression contributed by CNV, we also implemented an additional co-expression analysis with GRACE 32 — a recently proposed method which removes the influence of CNV before analysis. To implement the two analyses, we selected cervical cancer patients from TCGA who had both gene expression and copy number alteration, and finally kept 34 genes of 284 patients for GRACE and 48 genes of 296 patients for WGCNA. To apply GRACE, we quantile-normalized each gene expression and standardized each CNV.

To explore functional feature for those genes, we further performed functional enrichment analysis, including gene ontology (GO) and KEGG pathway, with DAVID 6.8 (https://david.ncifcrf.gov/) 33. Enrichment analysis allows us to validate our findings by determining functional annotations for those identified genes. In order to detect interaction and association, we also conducted protein-protein interaction analysis in terms of the Search Tool for the Retrieval of Interacting Genes/Proteins (STRING 11.0) database (https://string-db.org/) 34.

## Results

### New novel non-MHC genes associated with cervical cancer identified by TWAS

We here focus on the TWAS associations yielded from the six tissues that were considered to be most possibly relevant with cervical cancer (i.e. DGN and GTEx whole blood, EBV-transformed lymphocytes, ovary, uterus, breast and vagina in GTEx). Results based on all the available tissues in GTEx are relegated to Supplementary File; see also Figure S1 and Table S4. Specifically, a total of 20 distinct genes from 26 association pairs (FDR < 0.05) are discovered in terms of the six tissues that are relevant to cervical cancer (Table 1). Among those, both *HLA-DOB* and *RP11-384K6.2* display associations with cervical cancer in three tissues; 80.0% (16/20) are located in the MHC region (Chr 6: 25,000,000-34,000,000) and 70.0% (14/20) are protein coding genes.

In particular, four non-MHC genes (i.e. *WDR19*, *RP11-384K6.2* and *RP11-384K6.6* in Chr 4 and *ITSN* in Chr 21) are identified. Importantly, each of these four genes is not nearby (within 1Mb upstream and downstream) any previous GWAS index SNPs or associated genes (Figure S2-S4 and Table S1). Moreover, their expression levels are only weakly correlated with each other; for example, the Pearson's correlation coefficient of expression levels for all pairs of the four genes ranges from -0.123 (between *RP11.384K6.6* and *ITSN1*) to 0.304 (between *RP11.384K6.6* and *RP11.384K6.2*) in terms of 376 EUR individuals in the Geuvadis Project 19, implying that the four non-MHC genes are likely new novel genes involved in the risk of developing cervical cancer. Furthermore, the four genes are still associated with cervical cancer using conditional TWAS analysis after controlling for the strongest SNP within respective locus (Table S5), supporting their independent roles on the susceptibility of cervical cancer.

### Validation results of those associated genes in the EAS population

**Figure 3:**
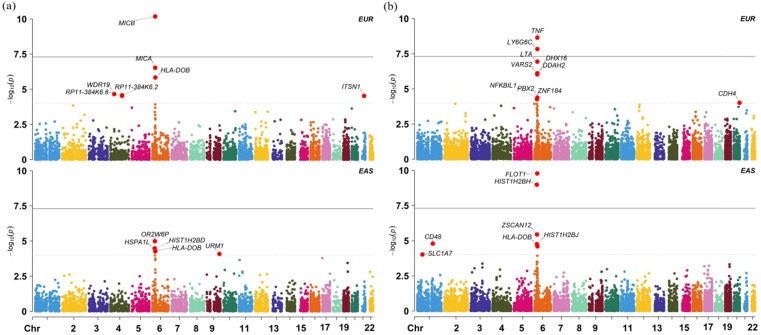
(a) Manhattan plot of TWAS results for the EAS and EUR ancestry cohorts in the blood tissue from GTEx; (b) Manhattan plot of TWAS results for the EAS and EUR ancestry cohorts in the blood tissue from DGN.

We further validate the associations discovered above using datasets from the EAS population and show the results in Table S6. In this validation analysis 18 out of 20 associated genes are found in the EAS population; among those, 4 genes (*p* = 1.47E-02 for *WDR19*, *p* = 2.65E-02 for *HLA-DOB*, *p* = 4.92E-02 for *MICB* and *p* = 1.78E-02 for *OR2B8P*) are identified to be also associated with cervical cancer at the nominal significance level of 0.05. Particularly, with regards to the four non-MHC genes, we observe that, besides *WDR19*, *ITSN1* is also detected to be marginally related to cervical cancer (*p* = 0.081) in the EAS population. However, we cannot replicate other associated genes in the EAS population. We also found many population-specific associated genes (Figure 3) from the GTEx and DGN whole blood with TWAS, such as *FLOT1* (*p* = 1.637E-10), *HISTIH2BH* (*p* = 1.037E-09).

### Results of gene co-expression network analysis

Taking both scale-free fit index and mean connectivity as reference, the soft-thresholding was determined to be 3 for WGCNA and GRACE. With WGCNA and using average linkage hierarchical clustering and module merging, all the genes were divided into five modules with different colors (Table S7). It means that most of the genes have high correlation with genes in the same module but weak correlation with genes in other modules. However, some genes (in grey) cannot be classified into any co-expression modules. With GRACE, the genes are divided into three modules (Table S7). The difference of co-expression genes between WGCNA and GRACE (TOM > 0.1) is shown in Figure S5-S6; and four potentially hub genes (e.g. *HLA.DQA1*, *HLA.DQB1*, *HLA.DRB1* and *SLAMF7*) are discovered by WGCNA. Of interest, all of them have co-expressed genes, such as *SLAMF7* and *LTA.*

### Gene enrichment analysis and Protein-protein interaction network

According to DAVID 6.8, those GWAS- or TWAS identified genes are enriched in nine GO terms (Table S8), some of which are significantly associated with cervical cancer. The significant KEGG pathways for cervical cancer are presented in Table S9. For example, it is shown that “type I diabetes mellitus” is most significantly enriched (FDR = 2.21E-9). The persistent high-risk HPV infection was associated with increased risk for cervical cancer and numbers of infiltrating T cells, particularly CD8 T cells, and the presentation of HPV E6/E7 epitopes were associated with improved prognosis 35. Other GO terms which obviously play important role in cervical cancer are also found, such as “MHC class II receptor activity”, “MHC class II protein complex” 36 and “peptide antigen binding” 37.

We further constructed the protein-protein interaction network to explore the biological function with STRING summary score above 0.4 (Figure S7). Each node represents a gene; the undirected link between two nodes is an edge, denoting the interaction between two genes. For example, strong interactions, such as *HLA-DRB1*, *TNF*, *LTA*, *HLA-DOB*, *LILRB1*, *MICA*, *HLA-C*, *HLA-B* and *MICB*, were previously detected to be associated with cervical cancer 38.

## Discussion

In the present study we have systematically evaluated the association of genetically predicted gene expression across various human tissues with the risk of cervical cancer. With extensively computational genetic analyses our results can provide substantial new insights into the understanding of genetics and etiology for cervical cancer. Importantly, we identified a set of associated genes in the EUR population including four promising new non-MHC genes (i.e. *WDR19*, *RP11-384K6.2*, *RP11-384K6.6* and *ITSN1*).

*RP11-384K6.6* is a type of IncRNA and discovered to be regulated by multiple differential miRNAs, such as *hsa-miR-16*, *hsa-miR-708* and *hsa-miR-486-5p* 39. Interestingly, Serum *miR-486-5p* can inhibit the *PTEN* expression and activate the oncogenic PI3K/Akt pathway in cervical cancer 40, and further stimulate cell proliferation, migration and invasion. *PTEN*, located at 10q23.3, is a tumor-suppressor gene and was identified to be associated with the risk of cervical cancer 41, 42, posing an important role in controlling cell growth, including promoting apoptosis, down-regulating adhesion and suppressing cell migration 43, 44. *RP11-384K6.2* is regulated by *hsa-miR-657*, which was found to have tumor-associated putative target genes that were associated with cervical cancer, including *CDC14B*, *TNFSF11* and *PTPN2* 45.

*WDR19* and *ITSN1* are also likely susceptibility genes of cervical cancer. Specifically, *WDR19* is the component of the IFT complex A (IFT-A). IFT-A modulates canonical Wnt/Wg-signalling, which was discovered to be involved in the risk of many cancers 46, 47. *ITSN1* belongs to the *ITSN* family and shares highly similar structures and functions with *ITSN2* 48-50. Importantly, *ITSN2* interacts with Eps8, the down-regulation of P53 and P21Waf1/Cip1 in cervical cancer 51.

We further validated our main finding in an independent EAS GWAS dataset 23. However, we only replicated the 4 genes including *WDR19*, *HLA-DOB*, *MICB* and *OR2B8P.* This failure may be due to the following reasons. First, we observe that substantial genetic heterogeneity exists in cervical cancer between the EUR and EAS populations. For example, the trans-ethnic genetic correlation of cervical cancer is estimated to be only *r_g_* = 0.038 (se = 0.015) with Linkage Disequilibrium Score regression 52 and 0.127 with Pearson's product-moment correlation, implying high ethnic difference in genetic foundation of cervical cancer. Indeed, we find many population-specific associated SNPs (Figure 2), such as rs3132468 (*p* = 0.095 in the EAS population but *p* = 6.56E-11 in the EUR population), rs2844484 (*p* = 1.12E-4 in the EAS population but *p* = 2.67E-9 in the EUR population) and rs3134954 (*p* = 0.014 in the EAS population but *p* = 1.24E-8 in the EUR). Second, except peripheral blood, we cannot access transcriptome profiles of other types of tissue (e.g. ovary) in our validation analysis. Given the distinct genetic architecture among various tissues 18 and diverse populations 53, it is unexpected that we cannot replicate genes that were discovered in non-peripheral-blood tissues. Note that, the two replicated newly associated genes (i.e. *WDR19* and *ITSN1*) in the EAS population were exactly also identified in blood in the EUR population. Third, the EAS transcriptome dataset has smaller sample size compared with the GTEx transcriptome datasets; for example, the average sample size of GTEx is about 200, two-fold higher than the EAS transcriptome dataset. In addition, the EAS cervical cancer GWAS also has smaller sample size (3,314 vs. 9,341) compared with the EUR cervical cancer GWAS. Therefore, we have reduced power when identifying associated genes in the EAS population.

In the subsequent functional analyses with GRACE, we showed that both *SLAMF7* and *LTA* were co-expressed in tumor samples consistent with the results by WGCNA. Moreover, we conducted GO enrichment and KEGG pathway analysis for both GWAS-identified and TWAS-identified genes, and found 9 GO terms and 16 pathways, respectively. We found that both *WDR19* and *ITSN1* were enriched in “*plasma membrane*”, which may be likely associated with cancer through its proteomics 54 or calcium channels 55. In addition, KEGG analysis found that type I diabetes mellitus was the most significantly enriched pathway, in line with the finding that diabetes is a well-known risk factor of cervical cancer 56-59.

Our multi-tissue transcriptome imputation approach has a number of advantages. Transcriptome imputation methods allow the study of genetically regulated gene expression without directly measuring expression data from an appropriate cell type in diseases. What's more, transcriptome imputation aggregates SNP-level associations to individual genes, reducing the multiple testing burden and increasing statistical power. Moreover, TWAS methods utilize eQTL information from eQTL databases with uniform sample collection and enable replication across the ancestry. However, our study has several limitations despite of these interesting results. The weights of *cis*-SNPs in TWAS were estimated with relatively small sample size for the external transcriptome reference data 16-20, which may underline the power of the TWAS analysis. As the sample size increases for relevant tissues, we expect that more potentially associated genes would be discovered. Besides, as it has shown that the leverage of gene expression reference panels from tissues that are less mechanistically related to diseases of interest can lead to bias and spuriously associated genes 24, the true mechanism and relevant tissues are still completely known although we carefully selected six tissues considered to be related to cervical cancer.

## Conclusion

This study discovers multiple candidate genes, especially four non-MHC genes, which may be causally associated with the risk of cervical cancer. However, further investigations with larger sample size are warranted to validate our findings in diverse populations.

## Supplementary Material

Supplementary figures and tables.

## Figures and Tables

**Table 1:** TWAS-identified genes associated with cervical cancer across the relevant tissues

Chr	position		Gene	Tissue	Gene type	*R*^2^	*z* value	*p* value	FDR
	Low	Up							
4	39,184,024	39,287,430	*WDR19*	GTEx WB	protein coding	0.049	4.20	2.20E-05	2.05E-02
4	119,553,429	119,555,914	*RP11-384K6.2*	EBV TL	pseudogene	0.100	4.21	2.69E-05	2.45E-02
4	119,553,429	119,555,914	*RP11-384K6.2*	ovary	pseudogene	0.108	4.20	2.60E-05	1.79E-02
4	119,553,429	119,555,914	*RP11-384K6.2*	GTEx WB	pseudogene	0.269	4.21	2.67E-05	2.05E-02
4	119,512,928	119,556,134	*RP11-384K6.6*	ovary	processed transcript	0.076	4.18	2.60E-05	1.79E-02
4	119,512,928	119,556,134	*RP11-384K6.6*	GTEx WB	processed transcript	0.251	4.24	2.96E-05	2.05E-02
21	35,014,706	35,272,165	*ITSN1*	GTEx WB	protein coding	0.077	-5.05	2.97E-05	2.05E-02
6	31,949,801	31,970,458	*C4A*	vagina	protein coding	0.094	-4.57	4.46E-07	4.75E-04
6	31,973,413	31,976,228	*CYP21A1P*	ovary	pseudogene	0.110	-4.94	4.93E-06	6.78E-03
6	31,694,815	31,698,394	*DDAH2*	DGN WB	protein coding	0.190	-4.92	7.71E-07	1.62E-03
6	30,620,896	30,640,814	*DHX16*	DGN WB	protein coding	0.001	-4.67	8.60E-07	1.62E-03
6	32,780,540	32,784,825	*HLA-DOB*	ovary	protein coding	0.600	-4.62	2.97E-06	6.78E-03
6	32,780,540	32,784,825	*HLA-DOB*	uterus	protein coding	0.500	-4.82	3.77E-06	8.17E-03
6	32,780,540	32,784,825	*HLA-DOB*	GTEx WB	protein coding	0.480	5.07	1.45E-06	2.67E-03
6	32,520,490	32,527,799	*HLA-DRB6*	vagina	pseudogene	0.451	-5.30	4.08E-07	4.75E-04
6	31,539,831	31,542,101	*LTA*	DGN WB	protein coding	0.107	-5.67	1.15E-07	4.05E-04
6	31,686,425	31,689,622	*LY6G6C*	DGN WB	protein coding	0.003	5.13	1.47E-08	7.74E-05
6	31,367,561	31,384,016	*MICA*	GTEx WB	protein coding	0.189	4.49	2.96E-07	8.18E-04
6	31,462,658	31,478,901	*MICB*	EBV TL	protein coding	0.148	6.53	7.22E-06	9.86E-03
6	31,462,658	31,478,901	*MICB*	GTEx WB	protein coding	0.339	3.88	6.56E-11	3.63E-07
6	28,021,006	28,021,943	*OR2B8P*	ovary	pseudogene	0.247	-3.87	1.03E-04	4.26E-02
6	28,083,406	28,084,329	*RP1-265C24.5*	ovary	pseudogene	0.075	4.13	1.08E-04	4.26E-02
6	31,926,857	31,937,532	*SKIV2L*	vagina	protein coding	0.180	-5.98	3.62E-05	2.57E-02
6	31,543,344	31,546,113	*TNF*	DGN WB	protein coding	0.082	4.91	2.27E-09	2.39E-05
6	30,876,019	30,894,236	*VARS2*	DGN WB	protein coding	0.371	-3.88	9.20E-07	1.62E-03
6	28,317,691	28,335,336	*ZKSCAN3*	ovary	protein coding	0.144	-4.18	1.03E-04	4.26E-02

Note: EBV TL: EBV transformed lymphocytes; WB: whole blood; DGN: Depression Genes and Networks. *R*^2^ shows the prediction accuracy of the *cis*-SNPs on gene expression in a tissue of GTEx.

## Abbreviations

TWAS: transcriptome-wide association study
GWAS: genome-wide association studies
HPV: human papillomavirus
SNP: single nucleotide polymorphisms
MHC: major histocompatibility complex
LD: linkage disequilibrium
eQTL: expression quantitative trait loci
FDR: false discover rate
TOM: topological overlap measurement
CNV: copy number variants
