# Supplementary material for: New novel non-MHC genes were identified for cervical cancer with an integrative analysis approach of transcriptome-wide association study
Source: J Cancer. 2021 Jan 1;12(3):840–8. doi: 10.7150/jca.47918 (PMC7778537; doi:10.7150/jca.47918)
Supplement: Supplementary file 1 — Supplementary figures and tables. [file jcav12p0840s1.pdf]

Supplementary Material

Determination of cervical cancer related tissues

To investigate related tissues for cervical cancer, we followed our previous work via PubMed retrieval [1] with the following specific strategy: “cervical cancer” [Title/Abstract] And (“Adipose” [Title/Abstract] or “Adrenal Gland” [Title/Abstract] or “Artery” [Title/Abstract] or “Brain” [Title/Abstract] or “EBV-transformed lymphocytes” [Title/Abstract] or “Skin - Transformed fibroblasts” [Title/Abstract] or “Colon” [Title/Abstract] or “Esophagus” [Title/Abstract] or “Heart” [Title/Abstract] or “Liver” [Title/Abstract] or “Lung” [Title/Abstract] or “Minor Salivary Gland” [Title/Abstract] or “Muscle - Skeletal” [Title/Abstract] or “Nerve - Tibial” [Title/Abstract] or “Ovary” [Title/Abstract] or “Pancreas” [Title/Abstract] or “Pituitary” [Title/Abstract] or “Skin” [Title/Abstract] or “Small Intestine” [Title/Abstract] or “Spleen” [Title/Abstract] or “Stomach” [Title/Abstract] or “Thyroid” [Title/Abstract] or “Uterus” [Title/Abstract] or “Vagina” [Title/Abstract] or “Whole Blood” [Title/Abstract]).

We obtained a total of 3,328 articles (until December 2019), of which 624 reported trait-tissues relevance. We further normalized this information by dividing the number of publications for a given tissue by the total number across all tissues. We selected gynecological tissues (e.g. vagina, ovary and uterus) to be the most relevant tissues based on the following lines of evidence. (1) the fluctuation of cyclin-dependent kinases (e.g. cdk2, cdk4, cdk6 and cdc2/cdk1), including their essential activating coenzymes, show in these gynecological cancers [2]. (2) Ovarian, cervix and endometrial cancer have the same mutations in genes *p53*, *BRCA1*, *DCC* and *PTEN* [3] and also represent the role of hypoxia inducible factor-1alpha [4], expression pattern of matrix metalloproteinase [5]. (3) Estrogen influences the physiological processes in breast, endometrium, ovary, cervix and vagina through its nuclear receptors ERa and ERb, and membrane receptor GPR30 [6]. (4) Cervix, uterus and vagina were affected by human papillomavirus (HPV). Whole blood was also selected to be a relevant tissue since the HPV-specific immune responses were detected in the lesions and peripheral blood of people with active and resolving

human papillomavirus-associated disease [7]. Overall, we finally chose six tissues (i.e. lymphocytes, vagina, ovary, uterus, breast and whole blood) to be the most relevant tissues for cervical cancer.

Associated genes identified by MetaXcan from all the tissues

With MetaXcan, we first performed a comprehensive association study between genetically predicted expression levels and cervical cancer to identified associated genes. Briefly, genes in 48 tissues (DGN and GTEx whole blood, and 47 other GTEx tissues; Table S2) were analyzed. Totally, 240 genes with false discover rate (FDR) less than 0.05 were discovered to be associated with cervical cancer.

After excluding male-specific tissues (i.e. prostate/testis), 213 associations (89 unique genes) were identified in almost the examined tissues except three (i.e. brain substantia nigra, breast mammary and stomach), with the number of associated genes in various tissues ranging from 11 (e.g. esophagus mucosa, transformed fibroblasts and pancreas) to 1 (e.g. artery coronary and uterus). Among all the identified genes (Table S4 and Figure S1), *HLA-DOB* has the highest frequency (16.0% = 34/213; FDR ranging from 1.55E-4 in pituitary to 4.70E-2 in liver), following by *RP11-384K6.6* (6.6% = 14/213; false discover rate [FDR] ranges from 8.70E-3 in no-sun exposed suprapubic skin to 4.65E-2 in nerve tibial) and *CYCSP34* (5.6% = 12/213; FDR ranges from 1.59E-3 in adipose subcutaneous to 4.40E-2 in esophagus muscularis). The majority of those TWAS-identified genes are located in Chr 6 (69.0% = 147/213), especially in the MHC region (Chr 6: 25,000,000-34,000,000) [8] (only two non-MHC associated genes in Chr 6). Functionally, most of the associated genes are protein coding genes (69.0%, 147/213) and processed transcript genes (6.6%, 14/213); and of those genes 23.9% (51/213) are pseudo genes with only one lincRNAs.

Note that, there are 20 unique TWAS-identified genes (a total of 26 associations) in the six cervical-cancer relevant tissues (Table 1); among those four are non-MHC genes located in Chr 4 and 21. Therefore, in the subsequent analysis we focus on those genes.

Table S1. SNPs and Genes that were reported to be associated with the risk of cervical cancer through GWAS.

PMID	Year	*N* (case/control)	Population	Region	SNPs	Position	Mapped Gene	*P*	Ref
23482656	2013	1,034/3,948	European	6p21.33	rs2516448	31,422,633	*MICA*	1.60E-18	[12]
23482656	2013	1,034/3,948	European	6p21.32	rs9272143	32,633,026	*HLA-DRB1*	9.30E-24	
23482656	2013	1,034/3,948	European	6p21.32	rs3117027	32,633,026	*HLA-DQA1*	4.90E-08	
23482656	2013	1,034/3,948	European	6p21.32	rs3117027	33,121,846	*HLA-DPB2*	1.00E-07	
23817570	2013	1,364/3,028	Han Chinese	4q12	rs13117307	55,885,574	*EXOC1*	3.00E-10	[13]
23817570	2013	1,364/3,028	Han Chinese	6p21.32	rs4282438	33,104,395	*HLA-DPB2*	5.00E-27	
23817570	2013	1,364/3,028	Han Chinese	17q21.1	rs8067378	39,895,095	*GSDMB*	9.00E-10	
23817570	2013	1,364/3,028	Han Chinese	6p21.32	rs9277952	33,236,497	*intergenic*	2.00E-09	
24700089	2014	226/186	Japanese	2q36.3	rs997363	227,644,742	*intergenic*	1.00E-06	[14]
24700089	2014	226/186	Japanese	7q11.23	rs7780883	77,433,395	*intergenic*	2.00E-06	
24700089	2014	226/186	Japanese	2q14.2	rs6726538	118,131,653	*intergenic*	3.00E-06	
24700089	2014	226/186	Japanese	18q22.3	rs8088832	72,259,522	*intergenic*	4.00E-06	
24700089	2014	226/186	Japanese	1q23.3	rs12068654	160,773,959	*intergenic*	5.00E-06	
24700089	2014	226/186	Japanese	16p13.2	rs4782151	9,232,128	*intergenic*	5.00E-06	
24700089	2014	226/186	Japanese	8p21.3	rs11985951	20,734,998	*intergenic*	6.00E-06	
24700089	2014	226/186	Japanese	8p23.3	rs6558578	2,021,565	*intergenic*	7.00E-06	
24700089	2014	226/186	Japanese	7q21.13	rs3899697	88,970,471	*ZNF804B*	9.00E-06	
27285765	2016	1,632/4,442	Swedish	6p21.3	rs73730372	32,616,804	*intergenic*	3.00E-19	[15]
28120872	2017	66/160	Han Chinese	4q34.3	rs6812281	179,391,655	*intergenic*	9.00E-09	[16]
28120872	2017	66/160	Han Chinese	14q32.11	rs1742101	90,698,719	*SNORA11B*	7.00E-06	
28120872	2017	66/160	Han Chinese	14q32.11	rs1742101	90,698,719	*CALM1*	7.00E-06	
28120872	2017	66/160	Han Chinese	14q32.11	rs1742101	90,698,719	*TTC7B*	7.00E-06	
28120872	2017	66/160	Han Chinese	14q32.11	rs1742101	90,698,719	*NRDE2*	7.00E-06	
28120872	2017	66/160	Han Chinese	14q32.11	rs1742101	90,698,719	*PSMC1*	7.00E-06	
28120872	2017	66/160	Han Chinese	14q32.11	rs1742101	90,698,719	*RPS18P2*	7.00E-06	
28120872	2017	66/160	Han Chinese	14q32.11	rs1742101	90,698,719	*RPS6KA5*	7.00E-06	

28120872	2017	66/160	Han Chinese	14q32.11	rs1742101	90,698,719	*RNU7- 30P*	7.00E-06	
28120872	2017	66/160	Han Chinese	14q32.11	rs1742101	90,698,719	*C14orf159*	7.00E-06	
28120872	2017	66/160	Han Chinese	16q23.3	rs1364121	83,706,502	*MIR3182*	3.00E-06	
28120872	2017	66/160	Han Chinese	16q23.3	rs1364121	83,706,502	*HSBP1*	3.00E-06	
28120872	2017	66/160	Han Chinese	16q23.3	rs1364121	83,706,502	*MLYCO*	3.00E-06	
28120872	2017	66/160	Han Chinese	16q23.3	rs1364121	83,706,502	*OSGIN1*	3.00E-06	
28120872	2017	66/160	Han Chinese	16q23.3	rs1364121	83,706,502	*NECAB2*	3.00E-06	
28120872	2017	66/160	Han Chinese	16q23.3	rs1364121	83,706,502	*SLC38A8*	3.00E-06	
28120872	2017	66/160	Han Chinese	16q23.3	rs1364121	83,706,502	*RNA5SP432*	3.00E-06	
28120872	2017	66/160	Han Chinese	16q23.3	rs1364121	83,706,502	*HSDL1*	3.00E-06	
28120872	2017	66/160	Han Chinese	16q23.3	rs1364121	83,706,502	*CDH13*	3.00E-06	
28120872	2017	66/160	Han Chinese	16q23.3	rs1364121	83,706,502	*ADAD2*	3.00E-06	
28120872	2017	66/160	Han Chinese	16q23.3	rs1364121	83,706,502	*TAF1C*	3.00E-06	
28120872	2017	66/160	Han Chinese	16q23.3	rs1364121	83,706,502	*DNAAF1*	3.00E-06	
28120872	2017	66/160	Han Chinese	16q23.3	rs1364121	83,706,502	*MBTPS1*	3.00E-06	
28120872	2017	66/160	Han Chinese	16q23.3	rs1364121	83,706,502	*MICB*	3.00E-06	
28806749	2017	2,866/6,481	European	6p21.33	rs3132461	31,512,891	*MICB*	2.00E-13	[17]
28806749	2017	2,866/6,481	European	6p21.33	rs3132461	31,512,891	*MCCD1*	2.00E-13	
28806749	2017	2,866/6,481	European	4p12	rs4396968	47,087,167	*GABRB1*	1.00E-06	
28806749	2017	2,866/6,481	European	7q34	rs2267681	139,841,136	*TBXAS1*	2.00E-06	
28806749	2017	2,866/6,481	European	8p23.1	rs56804039	8,523,519	*SGK223*	4.00E-06	
28806749	2017	2,866/6,481	European	8p23.1	rs56804039	8,523,519	*CLDN23*	4.00E-06	
28806749	2017	2,866/6,481	European	8q13.3	rs4738017	69,663,895	*SULF1*	7.00E-06	
28806749	2017	2,866/6,481	European	8q13.3	rs4738017	69,663,895	*SLCO5A1*	7.00E-06	
28806749	2017	2,866/6,481	European	13q14.11	rs9532669	40,927,414	*SUGT1P3*	2.00E-07	
28806749	2017	2,866/6,481	European	13q14.11	rs9532669	40,927,414	*ELF1*	2.00E-07	
28806749	2017	2,866/6,481	European	15q26.3	rs11637339	99,451,803	*LRRC28*	6.00E-06	
28806749	2017	2,866/6,481	European	15q26.3	rs11637339	99,451,803	*MEF2A*	6.00E-06	
28806749	2017	2,866/6,481	European	18q22.3	rs17087933	73,596,115	*LOC100505817*	2.00E-06	
28806749	2017	2,866/6,481	European	18q22.3	rs17087933	73,596,115	*FBXO15*	2.00E-06	

28806749	2017	2,866/6,481	European	6p21.33	rs2239704	31,572,364	*TNF*	6.00E-09	
28806749	2017	2,866/6,481	European	6p21.3	rs9271858	32,627,446	*MHC*	5.20E-15	
28806749	2017	2,866/6,481	European	6p21.32	rs9272143	32,633,026	*HLA-DRB1*	5.00E-15	
30412241	2017	2,866/6,481	Han Chinese and Japanese	6p21.32	rs6547598	-	*RN7SL251P*	5.00E-15	[18]
30412241	2017	2,866/6,481		6p21.32	rs59661306	91,087,644	*ADGRV1*	5.00E-15	
30412241	2017	2,866/6,481	Han Chinese and Japanese	6p21.32	rs7457728	54,380,269	*HPVC1*	5.00E-15	
30412241	2017	2,866/6,481		6p21.32	rs7457728	54,380,269	*LINC01445*	5.00E-15	
30412241	2017	2,866/6,481	Han Chinese and Japanese	6p21.32	rs7457728	54,380,269	*VSTM2A-OT1*	5.00E-15	
30412241	2017	2,866/6,481		6p21.32	rs7457728	54,380,269	*VSTM2A*	5.00E-15	
30412241	2017	2,866/6,481	Han Chinese and Japanese	6p21.32	rs115137622	33,143,124	*HLA-DPB2*	4.00E-12	
30412241	2017	2,866/6,481		6p21.32	rs8067378	39,895,095	*GSDMB*	4.00E-12	

Table S2. Summary information of tissues in GTEx data sets used in the our TWAS analysis.

Tissues	*N*	Gene numbers
Adipose subcutaneous	328	8,270
Adipose visceral (omentum)	273	6,594
Adrenal gland	146	4,608
Artery aorta	236	6,656
Artery coronary	128	3,507
Artery tibial	329	8,235
Brain amygdala	81	2,369
Brain anterior cingulate cortex (BA24)	102	3,308
Brain caudate (basal ganglia)	126	4,184
Brain cerebellar hemisphere	113	4,760
Brain cerebellum	137	6,103
Brain cortex	119	4,332
Brain frontal cortex (BA9)	104	3,607
Brain hippocampus	99	2,824
Brain hypothalamus	98	2,839
Brain nucleus accumbens (basal ganglia)	114	3,633
Brain putamen (basal ganglia)	98	3,178
Brain spinal cord (cervical c-1)	76	2,520
Brain substantia nigra	70	2,043
Breast mammary tissue	211	5,312
Blood EBV transformed lymphocytes	96	3,045
Skin transformed fibroblasts	256	7,354
Colon sigmoid	185	5,242
Colon transverse	210	5,614
Esophagus gastroesophageal junction	185	5,263
Esophagus mucosa	307	8,181
Esophagus muscularis	287	7,748
Heart atrial appendage	231	5,990
Heart left ventricle	233	5,382
Liver	134	3,355
Lung	333	7,968
Minor salivary gland	74	2,230
Muscle skeletal	421	7,521
Nerve tibial	305	9,444
Ovary	99	3,060
Pancreas	180	5,339
Pituitary	143	4,776
Prostate	114	3,268
Skin not sun exposed (Suprapubic)	285	7,641
Skin sun exposed (lower leg)	359	9,119
Small intestine terminal ileum	103	3,113
Spleen	119	4,615
Stomach	200	4,797
Testis	191	9,066
Thyroid	344	9,661
Uterus	82	2,434

Vagina	91	2,386
Whole blood (GTEx)	315	6,297
Whole blood (DGN)	922	12,719

Note: *N*: the total number of samples; Gene numbers: number of converged genes with heritability estimation.

Table S3. Relevant tissues for cervical cancer determined via PubMed search.

Tissues	*N* (%)
Blood EBV transformed lymphocytes	114 (18.27)
Vagina	112 (17.95)
Ovary	88 (14.10)
Uterus	67 (10.74)
Breast	53 (8.49)
Whole blood	46 (7.37)
Lung	39 (6.25)
Brain	25 (4.01)
Artery	21 (3.37)
Skin	14 (2.24)
Liver	11 (1.76)
Thyroid	5 (0.80)
Colon	4 (0.64)
Small intestine terminal ileum	4 (0.64)
Spleen	4 (0.64)
Muscle skeletal	3 (0.48)
Stomach	3 (0.48)
Adrenal gland	2 (0.32)
Esophagus	2 (0.32)
Heart	2 (0.32)
Pancreas	2 (0.32)
Pituitary	2 (0.32)
Adipose	1 (0.16)
Minor salivary gland	0 (0)
Nerve tibial	0 (0)

Table S4. TWAS-identified genes associated with cervical cancer using all the tissues in GTEx.

Chr	Position		Gene	Genotype	Tissue	R^2	z value	*p* value	FDR
	Low	Up							
2	70,377,012	70,475,747	*C2orf42*	protein coding	Lung	0.022	4.46	8.38E-06	9.78E-03
2	114,195,268	114,253,766	*CBWD2*	protein coding	Brain cerebellar hemisphere	0.285	-4.48	7.47E-06	8.05E-03
2	113,973,574	114,036,527	*PAX8*	protein coding	Cells transformed fibroblasts	0.374	-4.39	1.15E-05	1.50E-02
2	113,914,902	113,960,814	*PSD4*	protein coding	Adipose subcutaneous	0.232	4.38	1.20E-05	2.93E-02
2	152,266,397	152,364,527	*RIF1*	protein coding	Heart left ventricle	0.032	4.07	4.72E-05	4.48E-02
2	69,821,873	69,822,190	*RP11-77O7.1*	pseudogene	Thyroid	0.014	-4.41	1.02E-05	2.19E-02
4	119,558,111	119,559,179	*CICP16*	pseudogene	Adipose subcutaneous	0.071	4.20	2.63E-05	3.70E-02
4	47,937,994	48,018,689	*CNGA1*	protein coding	Artery coronary	0.142	-4.79	1.64E-06	2.52E-03
4	119,553,429	119,555,914	*RP11-384K6.2*	pseudogene	Brain hypothalamus	0.150	4.21	2.60E-05	2.18E-02
4	119,553,429	119,555,914	*RP11-384K6.2*	pseudogene	Cells EBV-transformed lymphocytes	0.100	4.20	2.69E-05	2.45E-02
4	119,553,429	119,555,914	*RP11-384K6.2*	pseudogene	Esophagus mucosa	0.215	4.10	4.20E-05	3.44E-02
4	119,553,429	119,555,914	*RP11-384K6.2*	pseudogene	Ovary	0.108	4.21	2.60E-05	1.79E-02
4	119,553,429	119,555,914	*RP11-384K6.2*	pseudogene	Pancreas	0.291	4.07	4.71E-05	3.54E-02
4	119,553,429	119,555,914	*RP11-384K6.2*	pseudogene	Skin not sun exposed suprapubic	0.162	4.21	2.60E-05	2.10E-02
4	119,553,429	119,555,914	*RP11-384K6.2*	pseudogene	Spleen	0.096	4.21	2.60E-05	2.69E-02
4	119,553,429	119,555,914	*RP11-384K6.2*	pseudogene	Whole blood	0.269	4.20	2.67E-05	2.05E-02
4	119,512,928	119,556,134	*RP11-384K6.6*	processed transcript	Adipose subcutaneous	0.301	4.10	4.13E-05	4.30E-02
4	119,512,928	119,556,134	*RP11-384K6.6*	processed transcript	Adipose visceral omentum	0.254	4.27	1.91E-05	2.06E-02
4	119,512,928	119,556,134	*RP11-384K6.6*	processed transcript	Artery aorta	0.183	4.20	2.68E-05	3.17E-02
4	119,512,928	119,556,134	*RP11-384K6.6*	processed transcript	Brain cerebellar hemisphere	0.085	4.21	2.60E-05	1.87E-02
4	119,512,928	119,556,134	*RP11-384K6.6*	processed transcript	Brain nucleus accumbens	0.126	4.01	6.16E-05	3.37E-02

					basal ganglia				
4	119,512,928	119,556,134	*RP11-384K6.6*	processed transcript	Brain spinal cord cervical c-1	0.183	3.96	7.53E-05	4.22E-02
4	119,512,928	119,556,134	*RP11-384K6.6*	processed transcript	Cells transformed fibroblasts	0.231	4.21	2.54E-05	2.76E-02
4	119,512,928	119,556,134	*RP11-384K6.6*	processed transcript	Colon sigmoid	0.124	4.12	3.77E-05	4.37E-02
4	119,512,928	119,556,134	*RP11-384K6.6*	processed transcript	Heart left ventricle	0.119	4.15	3.38E-05	4.01E-02
4	119,512,928	119,556,134	*RP11-384K6.6*	processed transcript	Muscle skeletal	0.270	4.06	4.84E-05	4.58E-02
4	119,512,928	119,556,134	*RP11-384K6.6*	processed transcript	Nerve tibial	0.270	4.24	2.21E-05	4.65E-02
4	119,512,928	119,556,134	*RP11-384K6.6*	processed transcript	Ovary	0.076	4.21	2.60E-05	1.79E-02
4	119,512,928	119,556,134	*RP11-384K6.6*	processed transcript	Skin not sun exposed suprapubic	0.195	4.47	7.71E-06	8.70E-03
4	119,512,928	119,556,134	*RP11-384K6.6*	processed transcript	Whole blood	0.251	4.18	2.96E-05	2.05E-02
4	39,184,024	39,287,430	*WDR19*	protein coding	Whole blood	0.049	4.24	2.20E-05	2.05E-02
5	441,645	443,258	*C5orf55*	protein coding	Pancreas	0.105	3.88	1.03E-05	4.89E-02
5	441,645	443,258	*C5orf55*	protein coding	Skin not sun exposed suprapubic	0.262	4.06	4.98E-05	3.38E-02
6	32,065,907	32,096,030	*ATF6B*	protein coding	Cells transformed fibroblasts	0.312	6.53	6.56E-11	4.30E-07
6	31,512,239	31,516,204	*ATP6V1G2*	protein coding	Brain amygdala	0.098	-5.98	2.27E-09	4.82E-06
6	31,606,805	31,620,482	*BAG6*	protein coding	Brain cerebellar hemisphere	0.249	-4.03	5.48E-05	2.92E-02
6	31,606,805	31,620,482	*BAG6*	protein coding	Esophagus gastroesophageal junction	0.222	4.85	1.22E-06	1.89E-03
6	32,936,437	32,949,282	*BRD2*	protein coding	Esophagus muscularis	0.077	4.07	4.67E-05	4.58E-02
6	26,440,700	26,453,643	*BTN3A3*	protein coding	Pancreas	0.117	-4.01	5.95E-05	3.54E-02
6	32,359,241	32,379,511	*BTNL2*	protein coding	Nerve tibial	0.106	4.84	1.27E-06	5.35E-03
6	31,949,801	31,970,458	*C4A*	protein coding	Brain amygdala	0.192	-5.33	9.63E-08	1.02E-04
6	31,949,801	31,970,458	*C4A*	protein coding	Brain cerebellum	0.214	-4.69	2.72E-06	4.97E-03
6	31,949,801	31,970,458	*C4A*	protein coding	Brain cortex	0.083	-4.57	4.89E-06	6.33E-03

6	31,949,801	31,970,458	*C4A*	protein coding	Brain putamen basal ganglia	0.116	-4.64	3.50E-06	2.50E-03
6	31,949,801	31,970,458	*C4A*	protein coding	Heart left ventricle	0.328	-4.54	5.59E-06	1.32E-02
6	31,949,801	31,970,458	*C4A*	protein coding	Lung	0.328	-4.67	2.95E-06	4.91E-03
6	31,949,801	31,970,458	*C4A*	protein coding	Muscle skeletal	0.215	4.38	1.21E-05	2.01E-02
6	31,949,801	31,970,458	*C4A*	protein coding	Skin not sun exposed suprapubic	0.391	-4.63	3.71E-06	5.03E-03
6	31,949,801	31,970,458	*C4A*	protein coding	Vagina	0.094	-5.05	4.46E-07	4.75E-04
6	31,982,539	32,003,195	*C4B*	protein coding	Brain caudate basal ganglia	0.074	-4.64	3.50E-06	4.36E-03
6	31,686,371	31,694,491	*C6orf25*	protein coding	Thyroid	0.019	-5.98	2.27E-09	1.94E-05
6	31,110,216	31,126,015	*CCHCR1*	protein coding	Cells transformed fibroblasts	0.142	-4.69	2.76E-06	6.03E-03
6	31,973,413	31,976,228	*CYP21A1P*	pseudogene	Artery tibial	0.294	-4.39	1.15E-05	1.67E-02
6	31,973,413	31,976,228	*CYP21A1P*	pseudogene	Ovary	0.110	-4.57	4.93E-06	6.78E-03
6	31,973,413	31,976,228	*CYP21A1P*	pseudogene	Skin not sun exposed suprapubic	0.227	-4.19	2.79E-05	2.10E-02
6	32,006,042	32,009,447	*CYP21A2*	protein coding	Adipose subcutaneous	0.189	4.17	3.05E-05	3.70E-02
6	32,006,042	32,009,447	*CYP21A2*	protein coding	Artery aorta	0.107	5.69	1.24E-08	7.33E-05
6	32,006,042	32,009,447	*CYP21A2*	protein coding	Artery tibial	0.131	5.18	2.16E-07	7.86E-04
6	32,006,042	32,009,447	*CYP21A2*	protein coding	Colon sigmoid	0.099	5.92	3.13E-09	1.45E-05
6	32,006,042	32,009,447	*CYP21A2*	protein coding	Esophagus mucosa	0.042	5.01	5.32E-07	3.84E-03
6	32,006,042	32,009,447	*CYP21A2*	protein coding	Lung	0.067	6.71	1.90E-11	1.33E-07
6	32,006,042	32,009,447	*CYP21A2*	protein coding	Pituitary	0.081	5.58	2.47E-08	1.05E-04
6	31,694,815	31,698,394	*DDAH2*	protein coding	DGN-WB	0.190	-4.94	7.71E-07	1.62E-03
6	31,694,815	31,698,394	*DDAH2*	protein coding	Muscle skeletal	0.033	-4.94	7.64E-07	2.53E-03
6	30,620,896	30,640,814	*DHX16*	protein coding	DGN-WB	0.000	-4.92	8.60E-07	1.62E-03
6	30,509,154	30,525,008	*GNL1*	protein coding	Skin sun exposed lower leg	0.016	6.53	6.56E-11	5.32E-07
6	26,839,263	26,924,333	*GUSBP2*	pseudogene	Brain spinal cord cervical c-1	0.181	3.91	9.36E-05	4.22E-02

6	31,165,537	31,171,745	*HCG27*	protein coding	Brain putamen basal ganglia	0.304	4.64	3.50E-06	2.50E-03
6	31,165,537	31,171,745	*HCG27*	protein coding	Skin not sun exposed suprapubic	0.229	4.76	1.92E-06	4.33E-03
6	27,106,073	27,114,619	*HIST1H2BK*	protein coding	Adipose visceral omentum	0.047	4.20	2.62E-05	2.17E-02
6	27,791,884	27,792,257	*HIST1H4J*	protein coding	Artery tibial	0.019	-4.14	3.40E-05	4.12E-02
6	31,321,649	31,324,965	*HLA-B*	protein coding	Brain caudate basal ganglia	0.068	6.69	2.30E-11	8.59E-08
6	31,321,649	31,324,965	*HLA-B*	protein coding	Esophagus muscularis	0.068	6.69	2.30E-11	1.58E-07
6	31,321,649	31,324,965	*HLA-B*	protein coding	Muscle skeletal	0.055	6.69	2.30E-11	1.52E-07
6	31,236,526	31,239,907	*HLA-C*	protein coding	Brain frontal cortex BA9	0.556	4.60	4.31E-06	4.64E-03
6	32,916,390	32,938,493	*HLA-DMA*	protein coding	Colon sigmoid	0.074	5.26	1.44E-07	3.33E-04
6	32,916,390	32,938,493	*HLA-DMA*	protein coding	Heart atrial appendage	0.147	4.69	2.68E-06	7.05E-03
6	32,780,540	32,784,825	*HLA-DOB*	protein coding	Adipose subcutaneous	0.408	-4.18	2.89E-05	3.70E-02
6	32,780,540	32,784,825	*HLA-DOB*	protein coding	Adipose visceral omentum	0.407	-4.25	2.13E-05	2.06E-02
6	32,780,540	32,784,825	*HLA-DOB*	protein coding	Adrenal Gland	0.312	-4.75	2.00E-06	7.14E-03
6	32,780,540	32,784,825	*HLA-DOB*	protein coding	Artery aorta	0.456	-4.70	2.66E-06	6.90E-03
6	32,780,540	32,784,825	*HLA-DOB*	protein coding	Artery tibial	0.498	-4.43	9.29E-06	1.67E-02
6	32,780,540	32,784,825	*HLA-DOB*	protein coding	Brain amygdala	0.367	-4.45	8.63E-06	6.12E-03
6	32,780,540	32,784,825	*HLA-DOB*	protein coding	Brain anterior cingulate cortex BA24	0.262	-4.84	1.32E-06	3.92E-03
6	32,780,540	32,784,825	*HLA-DOB*	protein coding	Brain caudate basal ganglia	0.481	-5.05	4.33E-07	8.08E-04
6	32,780,540	32,784,825	*HLA-DOB*	protein coding	Brain cerebellar hemisphere	0.553	-4.73	2.27E-06	6.26E-03
6	32,780,540	32,784,825	*HLA-DOB*	protein coding	Brain cerebellum	0.495	-4.60	4.24E-06	5.81E-03
6	32,780,540	32,784,825	*HLA-DOB*	protein coding	Brain frontal cortex ba9	0.605	-4.62	3.85E-06	4.64E-03
6	32,780,540	32,784,825	*HLA-DOB*	protein coding	Brain hypothalamus	0.532	-4.59	4.39E-06	8.84E-03
6	32,780,540	32,784,825	*HLA-DOB*	protein coding	Brain nucleus accumbens basal ganglia	0.501	-4.00	6.27E-05	3.37E-02

6	32,780,540	32,784,825	*HLA-DOB*	protein coding	Brain spinal cord cervical c-1	0.269	-4.94	7.91E-07	1.78E-03
6	32,780,540	32,784,825	*HLA-DOB*	protein coding	Cells transformed fibroblasts	0.585	-4.43	9.35E-06	1.50E-02
6	32,780,540	32,784,825	*HLA-DOB*	protein coding	Colon sigmoid	0.436	-4.65	3.28E-06	5.06E-03
6	32,780,540	32,784,825	*HLA-DOB*	protein coding	Esophagus gastroesophageal junction	0.414	-5.36	8.16E-08	3.78E-04
6	32,780,540	32,784,825	*HLA-DOB*	protein coding	Esophagus mucosa	0.407	-4.09	4.28E-05	3.44E-02
6	32,780,540	32,784,825	*HLA-DOB*	protein coding	Esophagus muscularis	0.533	-4.53	5.97E-06	1.02E-02
6	32,780,540	32,784,825	*HLA-DOB*	protein coding	Heart atrial appendage	0.328	-4.74	2.09E-06	7.05E-03
6	32,780,540	32,784,825	*HLA-DOB*	protein coding	Liver	0.185	-4.07	4.78E-05	4.70E-02
6	32,780,540	32,784,825	*HLA-DOB*	protein coding	Lung	0.527	-4.91	9.33E-07	2.18E-03
6	32,780,540	32,784,825	*HLA-DOB*	protein coding	Muscle skeletal	0.367	-4.79	1.67E-06	3.69E-03
6	32,780,540	32,784,825	*HLA-DOB*	protein coding	Nerve tibial	0.508	-5.30	1.19E-07	1.00E-03
6	32,780,540	32,784,825	*HLA-DOB*	protein coding	Ovary	0.600	-4.67	2.97E-06	6.78E-03
6	32,780,540	32,784,825	*HLA-DOB*	protein coding	Pancreas	0.329	-4.57	4.78E-06	1.14E-02
6	32,780,540	32,784,825	*HLA-DOB*	protein coding	Pituitary	0.278	-5.38	7.32E-08	1.55E-04
6	32,780,540	32,784,825	*HLA-DOB*	protein coding	Skin not sun exposed suprapubic	0.495	-4.43	9.29E-06	8.99E-03
6	32,780,540	32,784,825	*HLA-DOB*	protein coding	Skin sun exposed lower leg	0.523	-5.16	2.52E-07	6.82E-04
6	32,780,540	32,784,825	*HLA-DOB*	protein coding	Small intestine terminal ileum	0.078	-4.57	4.85E-06	4.47E-03
6	32,780,540	32,784,825	*HLA-DOB*	protein coding	Spleen	0.419	-4.54	5.73E-06	7.90E-03
6	32,780,540	32,784,825	*HLA-DOB*	protein coding	Thyroid	0.350	-4.92	8.78E-07	2.51E-03
6	32,780,540	32,784,825	*HLA-DOB*	protein coding	Uterus	0.500	-4.62	3.77E-06	8.17E-03
6	32,780,540	32,784,825	*HLA-DOB*	protein coding	Whole blood	0.480	-4.82	1.45E-06	2.67E-03
6	32,709,119	32,714,992	*HLA-DQA2*	protein coding	Brain cerebellar hemisphere	0.394	-4.01	6.11E-05	2.92E-02
6	32,709,119	32,714,992	*HLA-DQA2*	protein coding	Brain cerebellum	0.396	-4.01	6.14E-05	4.81E-02
6	32,709,119	32,714,992	*HLA-DQA2*	protein coding	Brain putamen basal ganglia	0.364	-4.01	6.11E-05	2.49E-02

6	32,709,119	32,714,992	*HLA-DQA2*	protein coding	Muscle skeletal	0.631	-4.32	1.59E-05	2.11E-02
6	32,546,546	32,557,625	*HLA-DRB1*	protein coding	Small intestine terminal ileum	0.082	5.06	4.10E-07	1.13E-03
6	32,485,120	32,498,064	*HLA-DRB5*	protein coding	Adipose visceral omentum	0.656	-5.09	3.60E-07	1.05E-03
6	32,485,120	32,498,064	*HLA-DRB5*	protein coding	Colon transverse	0.538	-4.94	7.63E-07	3.74E-03
6	32,485,120	32,498,064	*HLA-DRB5*	protein coding	Esophagus gastroesophageal junction	0.605	-4.90	9.43E-07	1.89E-03
6	32,485,120	32,498,064	*HLA-DRB5*	protein coding	Pancreas	0.645	-5.43	5.57E-08	2.65E-04
6	32,520,490	32,527,799	*HLA-DRB6*	pseudogene	Vagina	0.451	5.07	4.08E-07	4.75E-04
6	32,427,598	32,441,277	*HLA-DRB9*	pseudogene	Artery tibial	0.135	5.20	2.01E-07	7.86E-04
6	32,427,598	32,441,277	*HLA-DRB9*	pseudogene	Skin sun exposed lower leg	0.033	-4.94	7.95E-07	1.61E-03
6	31,349,851	31,350,065	*HLA-S*	pseudogene	Artery aorta	0.333	4.64	3.50E-06	6.90E-03
6	391,739	411,447	*IRF4*	protein coding	Adipose visceral omentum	0.058	-4.44	8.90E-06	1.29E-02
6	31,539,831	31,542,101	*LTA*	protein coding	DGN-WB	0.107	-5.30	1.15E-07	4.05E-04
6	31,637,944	31,641,553	*LY6G5B*	protein coding	Brain frontal cortex ba9	0.232	-5.73	1.01E-08	3.25E-05
6	31,637,944	31,641,553	*LY6G5B*	protein coding	Brain hippocampus	0.115	-5.20	2.01E-07	5.03E-04
6	31,637,944	31,641,553	*LY6G5B*	protein coding	Brain nucleus accumbens basal ganglia	0.141	-5.20	2.01E-07	6.47E-04
6	31,686,425	31,689,622	*LY6G6C*	protein coding	DGN-WB	0.003	-5.67	1.47E-08	7.74E-05
6	30,667,584	30,685,666	*MDC1*	protein coding	Cells transformed fibroblasts	0.017	-5.13	2.96E-07	9.70E-04
6	31,367,561	31,384,016	*MICA*	protein coding	Whole blood	0.189	5.13	2.96E-07	8.18E-04
6	31,462,658	31,478,901	*MICB*	protein coding	Adrenal gland	0.094	4.64	3.50E-06	7.14E-03
6	31,462,658	31,478,901	*MICB*	protein coding	Brain Cerebellum	0.604	6.69	2.30E-11	1.26E-07
6	31,462,658	31,478,901	*MICB*	protein coding	Brain putamen basal ganglia	0.277	5.66	1.48E-08	4.20E-05
6	31,462,658	31,478,901	*MICB*	protein coding	Cells EBV-transformed lymphocytes	0.148	4.49	7.22E-06	9.86E-03
6	31,462,658	31,478,901	*MICB*	protein coding	Cells transformed fibroblasts	0.473	4.12	3.85E-05	3.15E-02

6	31,462,658	31,478,901	*MICB*	protein coding	Liver	0.185	4.53	6.04E-06	1.78E-02
6	31,462,658	31,478,901	*MICB*	protein coding	Minor salivary gland	0.081	4.24	2.22E-05	2.24E-02
6	31,462,658	31,478,901	*MICB*	protein coding	Spleen	0.263	5.20	2.01E-07	4.16E-04
6	31,462,658	31,478,901	*MICB*	protein coding	Whole blood	0.339	6.53	6.56E-11	3.63E-07
6	31,707,725	31,732,622	*MSH5*	protein coding	Esophagus gastroesophageal junction	0.053	4.64	3.50E-06	4.05E-03
6	32,162,620	32,191,844	*NOTCH4*	protein coding	Brain spinal cord cervical c-1	0.128	-4.79	1.63E-06	1.84E-03
6	28,021,006	28,021,943	*OR2B8P*	pseudogene	Adrenal gland	0.159	4.04	5.39E-05	4.40E-02
6	28,021,006	28,021,943	*OR2B8P*	pseudogene	Brain cortex	0.143	4.09	4.41E-05	4.28E-02
6	28,021,006	28,021,943	*OR2B8P*	pseudogene	Ovary	0.247	3.88	1.03E-05	4.26E-02
6	32,116,136	32,122,150	*PRRT1*	protein coding	Thyroid	0.089	5.20	2.01E-07	8.62E-04
6	32,146,131	32,151,930	*RNF5*	protein coding	Spleen	0.048	-5.23	1.66E-07	4.16E-04
6	28,083,406	28,084,329	*RP1-265C24.5*	pseudogene	Brain amygdala	0.072	-4.05	5.03E-05	2.67E-02
6	28,083,406	28,084,329	*RP1-265C24.5*	pseudogene	Brain frontal cortex ba9	0.142	-4.04	5.42E-05	3.95E-02
6	28,083,406	28,084,329	*RP1-265C24.5*	pseudogene	Brain nucleus accumbens basal ganglia	0.459	-4.04	5.39E-05	3.37E-02
6	28,083,406	28,084,329	*RP1-265C24.5*	pseudogene	Brain putamen basal ganglia	0.057	-4.21	2.56E-05	1.21E-02
6	28,083,406	28,084,329	*RP1-265C24.5*	pseudogene	Ovary	0.075	-3.87	1.08E-05	4.26E-02
6	28,234,788	28,245,974	*RP5-874C20.3*	pseudogene	Brain caudate basal ganglia	0.201	-4.15	3.29E-05	3.07E-02
6	28,234,788	28,245,974	*RP5-874C20.3*	pseudogene	Brain hippocampus	0.128	-3.94	8.21E-05	4.11E-02
6	28,234,788	28,245,974	*RP5-874C20.3*	pseudogene	Muscle skeletal	0.133	-4.10	4.18E-05	4.58E-02
6	31,926,857	31,937,532	*SKIV2L*	protein coding	Esophagus muscularis	0.535	5.56	2.69E-08	9.22E-05
6	31,926,857	31,937,532	*SKIV2L*	protein coding	Vagina	0.180	4.13	3.62E-05	2.57E-02
6	33,168,222	33,172,216	*SLC39A7*	protein coding	Brain frontal cortex ba9	0.063	-4.01	6.11E-05	3.95E-02
6	31,981,047	31,981,564	*STK19P*	pseudogene	Artery aorta	0.144	4.07	4.69E-05	4.62E-02
6	31,981,047	31,981,564	*STK19P*	pseudogene	Brain cerebellar hemisphere	0.116	4.22	2.43E-05	1.87E-02

6	31,981,047	31,981,564	*STK19P*	pseudogene	Brain putamen basal ganglia	0.159	4.29	1.78E-05	1.02E-02
6	31,981,047	31,981,564	*STK19P*	pseudogene	Esophagus mucosa	0.350	4.40	1.08E-05	2.60E-02
6	31,981,047	31,981,564	*STK19P*	pseudogene	Small intestine terminal ileum	0.154	4.14	3.50E-05	1.94E-02
6	32,781,544	32,806,599	*TAP2*	protein coding	Adipose subcutaneous	0.223	5.06	4.10E-07	1.59E-03
6	32,781,544	32,806,599	*TAP2*	protein coding	Adipose visceral omentum	0.146	5.35	8.87E-08	5.15E-04
6	32,781,544	32,806,599	*TAP2*	protein coding	Cells transformed fibroblasts	0.366	4.08	4.53E-05	3.30E-02
6	32,781,544	32,806,599	*TAP2*	protein coding	Liver	0.041	4.14	3.50E-05	4.70E-02
6	32,781,544	32,806,599	*TAP2*	protein coding	Lung	0.130	4.07	4.67E-05	4.67E-02
6	31,543,344	31,546,113	*TNF*	protein coding	DGN-WB	0.082	-5.98	2.27E-09	2.39E-05
6	31,976,391	31,980,249	*TNXA*	pseudogene	Brain hippocampus	0.174	4.61	4.08E-06	5.10E-03
6	30,876,019	30,894,236	*VARS2*	protein coding	DGN-WB	0.371	4.91	9.20E-07	1.62E-03
6	30,876,019	30,894,236	*VARS2*	protein coding	Minor salivary gland	0.160	6.69	2.30E-11	4.64E-08
6	31,255,287	31,256,741	*WASF5P*	pseudogene	Brain cerebellum	0.177	-5.13	2.96E-07	8.10E-04
6	160,146,617	160,177,351	*WTAP*	protein coding	Nerve tibial	0.269	4.37	1.22E-05	3.44E-02
6	28,317,691	28,335,336	*ZKSCAN3*	protein coding	Ovary	0.144	-3.88	1.03E-05	4.26E-02
6	28,048,753	28,057,341	*ZNF165*	protein coding	Heart left ventricle	0.038	-4.16	3.11E-05	4.01E-02
6	27,325,604	27,339,304	*ZNF204P*	pseudogene	Adrenal gland	0.055	4.04	5.37E-05	4.40E-02
6	27,325,604	27,339,304	*ZNF204P*	pseudogene	Esophagus mucosa	0.024	4.02	5.71E-05	4.13E-02
6	35,226,686	35,263,762	*ZNF76*	protein coding	Skin not sun exposed suprapubic	0.015	-4.65	3.38E-06	5.03E-03
8	8,314,419	8,318,847	*CTA-398F10.2*	lincRNA	Esophagus mucosa	0.152	-4.13	3.56E-05	3.44E-02
8	128,959,126	128,960,591	*TMEM75*	protein coding	Esophagus mucosa	0.014	4.30	1.73E-05	3.13E-02
8	40,388,109	40,755,352	*ZMAT4*	protein coding	Artery tibial	0.152	4.09	4.24E-05	4.41E-02
9	27,546,544	27,573,864	*C9orf72*	protein coding	Esophagus mucosa	0.115	4.11	3.92E-05	3.44E-02
9	131,857,073	131,873,468	*CRAT*	protein coding	Cells transformed fibroblasts	0.166	4.18	2.95E-05	2.76E-02
9	134,378,289	134,399,193	*POMT1*	protein coding	Esophagus mucosa	0.058	4.50	6.73E-06	2.43E-02

10	75,668,935	75,677,255	*PLAU*	protein coding	Esophagus mucosa	0.024	-4.10	4.09E-05	3.44E-02
11	95,556,681	95,557,336	*AP001877.1*	pseudogene	Brain cerebellar hemisphere	0.444	4.53	5.98E-06	8.05E-03
11	46,958,240	47,185,936	*C11orf49*	protein coding	Esophagus muscularis	0.022	4.38	1.18E-05	1.61E-02
11	46,958,240	47,185,936	*C11orf49*	protein coding	Pancreas	0.133	3.98	6.82E-05	3.60E-02
11	47,608,198	47,610,746	*FAM180B*	protein coding	Brain cerebellum	0.076	-4.12	3.74E-05	3.41E-02
11	46,878,419	46,940,193	*LRP4*	protein coding	Spleen	0.049	4.10	4.07E-05	3.37E-02
11	46,402,306	46,405,375	*MDK*	protein coding	Esophagus mucosa	0.025	3.97	7.28E-05	4.78E-02
11	47,199,076	47,207,994	*PACSIN3*	protein coding	Cells transformed fibroblasts	0.052	3.99	6.68E-05	4.38E-02
13	41,437,735	41,438,038	*CYCSP34*	pseudogene	Adipose subcutaneous	0.088	5.05	4.35E-07	1.59E-03
13	41,437,735	41,438,038	*CYCSP34*	pseudogene	Adipose visceral omentum	0.050	4.51	6.39E-06	1.24E-02
13	41,437,735	41,438,038	*CYCSP34*	pseudogene	Artery aorta	0.067	4.51	6.39E-06	9.44E-03
13	41,437,735	41,438,038	*CYCSP34*	pseudogene	Artery tibial	0.178	4.51	6.39E-06	1.55E-02
13	41,437,735	41,438,038	*CYCSP34*	pseudogene	Brain anterior cingulate cortex BA24	0.131	4.43	9.58E-06	1.42E-02
13	41,437,735	41,438,038	*CYCSP34*	pseudogene	Brain hippocampus	0.265	4.15	3.38E-05	2.82E-02
13	41,437,735	41,438,038	*CYCSP34*	pseudogene	Brain hypothalamus	0.333	4.49	7.05E-06	8.84E-03
13	41,437,735	41,438,038	*CYCSP34*	pseudogene	Brain nucleus accumbens basal ganglia	0.328	4.28	1.84E-05	2.95E-02
13	41,437,735	41,438,038	*CYCSP34*	pseudogene	Brain putamen basal ganglia	0.117	4.74	2.10E-06	2.50E-03
13	41,437,735	41,438,038	*CYCSP34*	pseudogene	Esophagus muscularis	0.088	4.12	3.85E-05	4.40E-02
13	41,437,735	41,438,038	*CYCSP34*	pseudogene	Pancreas	0.071	4.33	1.52E-05	2.41E-02
13	41,437,735	41,438,038	*CYCSP34*	pseudogene	Small intestine terminal ileum	0.152	4.81	1.49E-06	2.07E-03
16	30,844,947	30,906,281	*BCL7C*	protein coding	Skin sun exposed lower leg	0.020	4.30	1.69E-05	2.28E-02
16	31,439,052	31,439,967	*COX6A2*	protein coding	Pancreas	0.038	-4.04	5.36E-05	3.54E-02
16	30,006,615	30,017,114	*INO80E*	protein coding	Pancreas	0.134	4.02	5.87E-05	3.54E-02
16	30,960,387	30,967,782	*ORAI3*	protein coding	Adipose visceral omentum	0.021	-4.07	4.68E-05	3.40E-02

16	30,773,066	30,787,628	*RNF40*	protein coding	Pancreas	0.060	4.19	2.81E-05	3.34E-02
21	35,014,706	35,272,165	*ITSN1*	protein coding	Whole blood	0.077	-4.18	2.97E-05	2.05E-02
21	47,706,251	47,717,665	*C2orf42*	protein coding	Brain cerebellar hemisphere	0.371	4.01	6.07E-05	2.92E-02

Note: Chr: Chromosome.

Table S5. Estimated FDR of the four TWAS-identified genes after adjusting for the top GWAS SNP in the loci.

Chr	Gene	Tissue	FDR_0	Top SNP	FDR_1
4	*WDR19*	GTEx WB	2.05E-02	rs4833103	2.31E-02
4	*RP11-384K6.2*	EBV TL	2.45E-02	rs7689541	2.48E-02
4	*RP11-384K6.2*	ovary	1.79E-02	rs7689541	1.79E-02
4	*RP11-384K6.2*	GTEx WB	2.05E-02	rs7689541	2.05E-02
4	*RP11-384K6.6*	ovary	1.79E-02	rs7689541	1.79E-02
4	*RP11-384K6.6*	GTEx WB	2.05E-02	rs7689541	2.05E-02
21	*ITSN1*	GTEx WB	2.05E-02	rs1125036	3.92E-02

Note: FDR_0 and FDR_1 are the FDR values estimated before or after the adjustment of the top SNP, respectively. EBV TL: EBV transformed lymphocytes; WB: whole blood.

Table S6. Validated the TWAS-identified genes in East Asian GWAS across the Peripheral blood

Chr	position		Gene	Gene type	*p*	$eSNP_1$		$eSNP_2$	
	Low	Up				eSNP	*p*	eSNP	*p*
4	39,184,024	39,287,430	*WDR19*	protein coding	1.47E-02	rs2066789	8.32E-02	rs12504238	2.74E-08
4	119,553,429	119,555,914	*RP11-384K6.2*	pseudogene	3.14E-01	rs35102067	1.60E-02	rs78454710	7.41E-05
4	119,512,928	119,556,134	*RP11-384K6.6*	processed transcript	2.71E-01	rs35102067	1.60E-02	rs78454710	7.41E-05
21	35,014,706	35,272,165	*ITSN1*	protein coding	8.13E-02	rs7280640	2.35E-02	rs8130507	5.78E-19
6	31,949,801	31,970,458	*C4A*	protein coding	8.94E-02	rs112395152	1.30E-04	rs12614	3.11E-12
6	31,973,413	31,976,228	*CYP21A1P*	pseudogene	5.66E-02	rs6450	1.80E-02	rs391165	1.11E-08
6	31,694,815	31,698,394	*DDAH2*	protein coding	8.34E-02	rs111541859	2.04E-04	rs1144708	4.41E-17
6	30,620,896	30,640,814	*DHX16*	protein coding	-	-	-	-	-
6	32,780,540	32,784,825	*HLA-DOB*	protein coding	2.65E-02	rs4148871	4.46E-05	rs241442	8.41E-18
6	32,520,490	32,527,799	*HLA-DRB6*	pseudogene	5.45E-02	rs535852	4.21E-02	rs535852	1.73E-13
6	31,539,831	31,542,101	*LTA*	protein coding	1.20E-01	rs2523504	1.28E-06	rs3132452	2.36E-13
6	31,686,425	31,689,622	*LY6G6C*	protein coding	9.17E-02	rs111541859	2.04E-04	rs1144708	4.41E-17
6	31,367,561	31,384,016	*MICA*	protein coding	3.72E-01	rs9468975	2.10E-01	rs2442719	2.62E-03
6	31,462,658	31,478,901	*MICB*	protein coding	4.92E-02	rs2523504	1.28E-06	rs141831504	8.23E-13
6	28,021,006	28,021,943	*OR2B8P*	pseudogene	1.78E-02	rs74505854	1.55E-04	rs10484402	2.36E-08
6	28,083,406	28,084,329	*RP1-265C24.5*	pseudogene	5.39E-02	rs7747772	1.43E-04	rs149956	1.36E-07
6	31,926,857	31,937,532	*SKIV2L*	protein coding	1.18E-01	rs117326768	1.29E-04	rs12614	3.11E-12
6	31,543,344	31,546,113	*TNF*	protein coding	1.21E-01	rs2523504	1.28E-06	rs3132452	2.36E-13
6	30,876,019	30,894,236	*VARS2*	protein coding	-	-	-	-	-
6	28,317,691	28,335,336	*ZKSCAN3*	protein coding	1.42E-01	rs9468335	4.45E-05	rs16893947	5.32E-10

Note: $eSNP_1$: SNP with the minimum *p* value in East Asian cervical cancer GWAS; $eSNP_2$: SNP with the minimum *p* value in the Peripheral blood.

Table S7. Highly interconnected genes in co-expression modules with WGCNA and GRACE adjusted for the effects of SCNA.

WGCNA		GRACE	
Module	Genes	Module	Genes
blue	*MEF2A*	blue	*SLAMF7*
	EXOC1		*TBXAS1*
	ELF1		*LTA*
	CYP21A1P	brown	*VARS2*
	C4A		*SKIV2L*
brown	*SLAMF7*		*DHX16*
	TBXAS1	turquoise	*MEF2A*
	LTA		*EXOC1*
	HLA.DPA3		*ELF1*
green	*HLA.C*		*C4A*
	HCP5		
	HLA.B		
turquoise	*INSIG2*		
	TTC7B		
	HLA.DQB1		
	HLA.DRB1		
	HLA.DQA1		
	HLA.DPB2		
	HLA.DRB6		
	HLA.DOB		
yellow	*VARS2*		
	SKIV2L		
	DHX16		

Table S8. Functional term enrichment analysis by combining both TWAS-identified and GWAS-identified genes.

GO ID	GO term	Genes	FDR
GO:0019882	antigen processing and presentation	*HLA-DQB1, MICB, MICA, HLA-DRB1, HLA-C, HLA-B, HLA-DQA1*	3.83E-06
GO:0032395	MHC class II receptor activity	*HLA-DQB1, HLA-DRB1, HLA-C, HLA-DOB, HLA-DQA1*	2.01E-05
GO:0042613	MHC class II protein complex	*HLA-DQB1, HLA-DRB1, HLA-C, HLA-DOB, HLA-DQA1*	1.35E-04
GO:0042605	peptide antigen binding	*HLA-DQB1, HLA-DRB1, HLA-C, HLA-B, HLA-DQA1*	2.96E-04
GO:0071556	integral component of lumenal side of endoplasmic reticulum membrane	*HLA-DQB1, HLA-DRB1, HLA-C, HLA-B, HLA-DQA1*	4.35E-04
GO:0012507	ER to Golgi transport vesicle membrane	*HLA-DQB1, HLA-DRB1, HLA-C, HLA-B, HLA-DQA1*	4.79E-03
GO:0005886	plasma membrane	*HLA-DQB1, MICB, TNF, MICA, C4A, HLA-DRB1, GSDMB, GABRB1, LY6G6C, TTC7B, HLA-C, HLA-B, SLAMF7, ITSN1, HLA-DQA1, CLDN23, OR2B8P, CDH13, WDR19, ADGRV1, SULF1, LTA, EXOC1*	5.36E-03
GO:0002504	antigen processing and presentation of peptide or polysaccharide antigen via MHC class II	*HLA-DQB1, HLA-DRB1, HLA-DOB, HLA-DQA1*	1.05E-02
GO:0060333	interferon-gamma-mediated signaling pathway	*HLA-DQB1, HLA-DRB1, HLA-C, HLA-B, HLA-DQA1*	2.96E-02

Note: Gene ontology (GO) enrichment analysis was performed by using the DAVID 6.8 (https://david.ncifcrf.gov/).

Table S9. KEGG pathway analysis for both the TWAS-identified and GWAS-identified genes.

Pathway names	Genes	FDR
Type I diabetes mellitus	*HLA-DQB1*, *TNF*, *HLA-DRB1*, *HLA-C*, *HLA-B*, *HLA-DOB*, *HLA-DQA1*, *LTA*	2.21E-09
Graft-versus-host disease	*HLA-DQB1*, *TNF*, *HLA-DRB1*, *HLA-C*, *HLA-B*, *HLA-DOB*, *HLA-DQA1*	6.27E-08
Allograft rejection	*HLA-DQB1*, *TNF*, *HLA-DRB1*, *HLA-C*, *HLA-B*, *HLA-DOB*, *HLA-DQA1*	1.31E-07
Antigen processing and presentation	*HLA-DQB1*, *TNF*, *HLA-DRB1*, *HLA-C*, *HLA-B*, *HLA-DOB*, *HLA-DQA1*	1.17E-05
Herpes simplex infection	*HLA-DQB1*, *TNF*, *HLA-DRB1*, *HLA-C*, *HLA-B*, *HLA-DOB*, *HLA-DQA1*, *LTA*	8.43E-05
Autoimmune thyroid disease	*HLA-DQB1*, *HLA-DRB1*, *HLA-C*, *HLA-B*, *HLA-DOB*, *HLA-DQA1*	8.93E-05
Viral myocarditis	*HLA-DQB1*, *HLA-DRB1*, *HLA-C*, *HLA-B*, *HLA-DOB*, *HLA-DQA1*	1.43E-04
Cell adhesion molecules (CAMs)	*HLA-DQB1*, *HLA-DRB1*, *HLA-C*, *HLA-B*, *HLA-DOB*, *CLDN23*, *HLA-DQA1*	5.05E-04
Asthma	*HLA-DQB1*, *TNF*, *HLA-DRB1*, *HLA-DOB*, *HLA-DQA1*	5.54E-04
HTLV-I infection	*HLA-DQB1*, *TNF*, *HLA-DRB1*, *HLA-C*, *HLA-B*, *HLA-DOB*, *HLA-DQA1*, *LTA*	7.96E-04
Staphylococcus aureus infection	*HLA-DQB1*, *C4A*, *HLA-DRB1*, *HLA-DOB*, *HLA-DQA1*	6.18E-03
Systemic lupus erythematosus	*HLA-DQB1*, *TNF*, *C4A*, *HLA-DRB1*, *HLA-DOB*, *HLA-DQA1*	1.03E-02
Inflammatory bowel disease (IBD)	*HLA-DQB1*, *TNF*, *HLA-DRB1*, *HLA-DOB*, *HLA-DQA1*	1.22E-02
Phagosome	*HLA-DQB1*, *HLA-DRB1*, *HLA-C*, *HLA-B*, *HLA-DOB*, *HLA-DQA1*	1.78E-02
Leishmaniasis	*HLA-DQB1*, *TNF*, *HLA-DRB1*, *HLA-DOB*, *HLA-DQA1*	1.85E-02
Rheumatoid arthritis	*HLA-DQB1*, *TNF*, *HLA-DRB1*, *HLA-DOB*, *HLA-DQA1*	4.34E-02

Note: KEGG pathway was performed by using the DAVID 6.8 (https://david.ncifcrf.gov/).

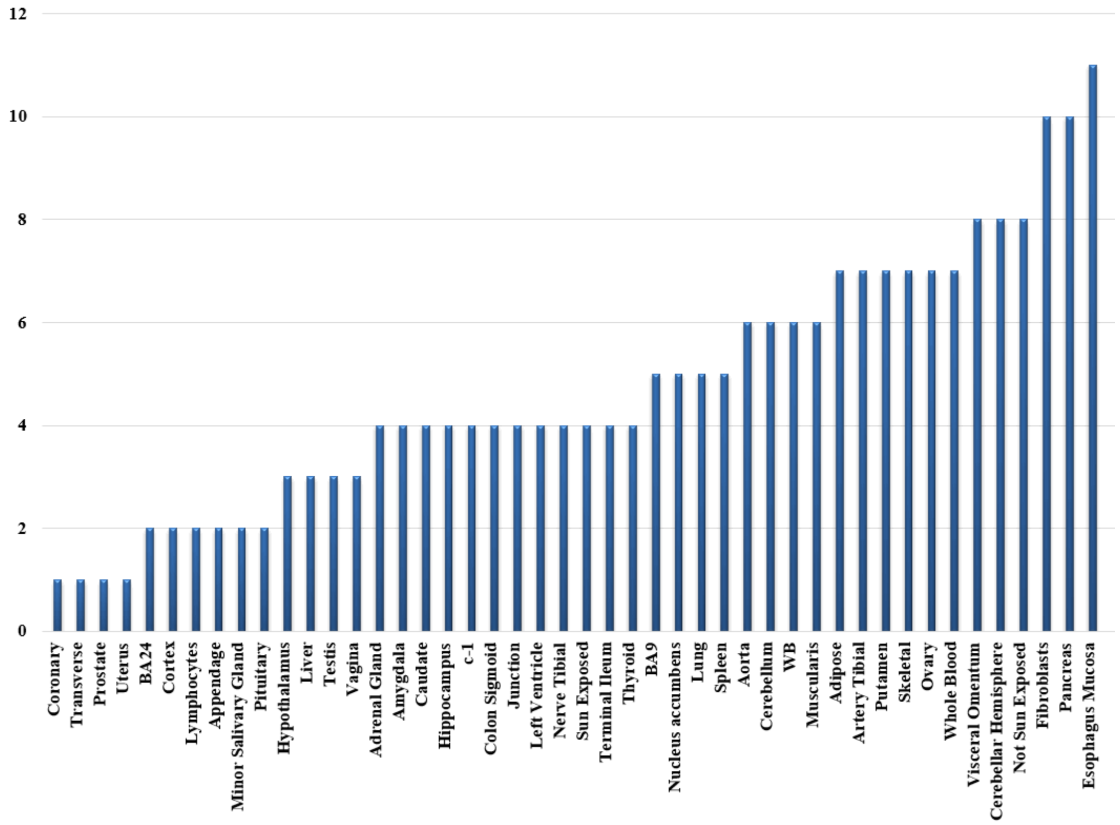

Figure S1. The number of associated genes in various GTEx tissues using MetaXcan. Coronary: Artery coronary; Transverse: Colon transverse; BA24: Brain anterior cingulate cortex BA24; Cortex: Brain cortex; Lymphocytes: Cells EBV transformed lymphocytes; Appendage: Heart atrial appendage; Hypothalamus: Brain hypothalamus; Amygdala: Brain amygdala; Caudate: Brain caudate basal ganglia; Hippocampus: Brain hippocampus; c-1: Brain spinal cord cervical c-1; Junction: Esophagus gastroesophageal junction; Left Ventricle: Heart left ventricle; Sun Exposed: Skin sun exposed lower leg; Terminal Ileum: Small intestine terminal ileum; BA9: Brain frontal cortex ba9; Nucleus accumbens: Brain nucleus accumbens basal ganglia; Aorta: Artery aorta; Cerebellum: Brain cerebellum; WB: DGN WB; Muscularis: Esophagus muscularis; Adipose: Adipose subcutaneous; Putamen: Brain putamen basal ganglia; Skeletal: Muscle skeletal; Visceral Omentum: Adipose visceral omentum; Cerebellar Hemisphere: Brain cerebellar hemisphere; Not Sun Exposed: Skin not sun exposed suprapubic; Fibroblasts: Cells transformed fibroblasts.

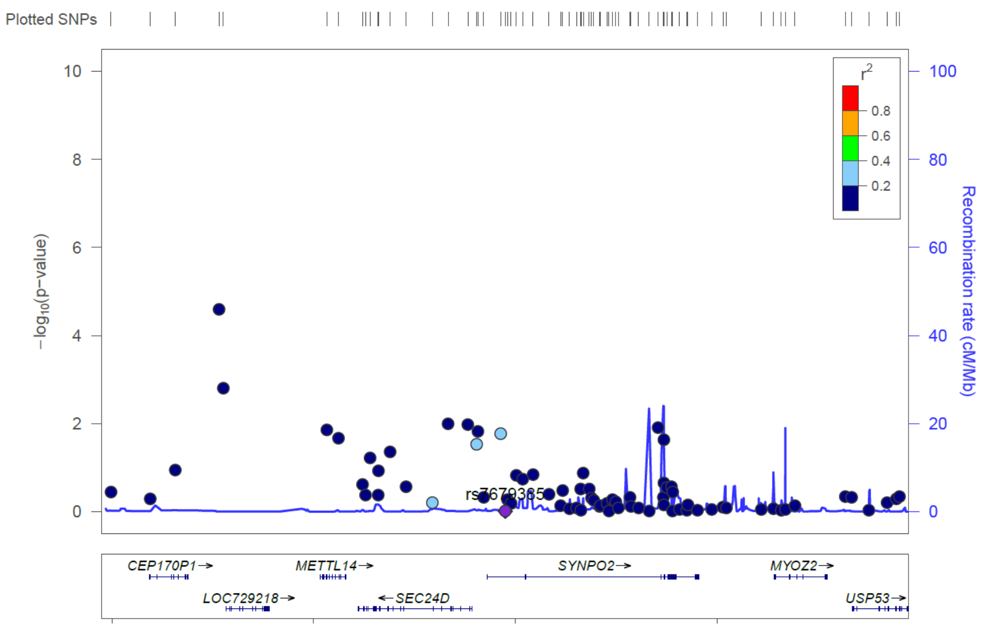

Figure S2. Gene position of the *RP11-384K6.2* and *RP11-384K6.6*.

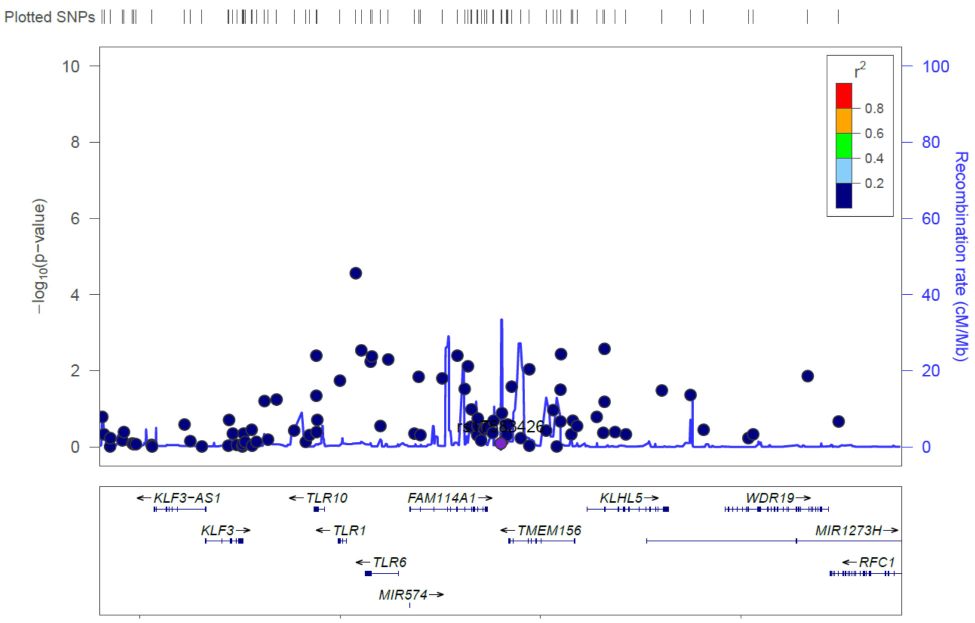

Figure S3. Gene position of the *WDR19*.

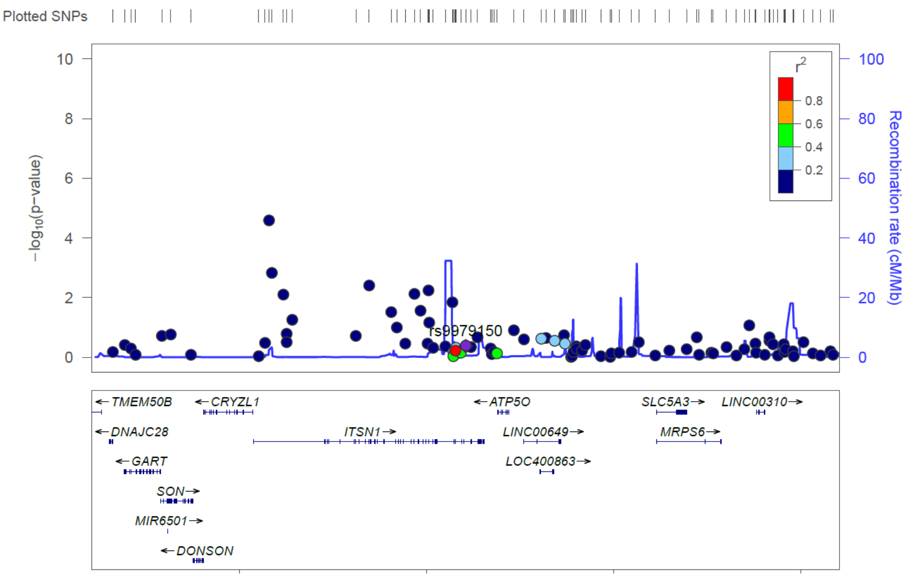

Figure S4. Gene position of the *ITSN1*.

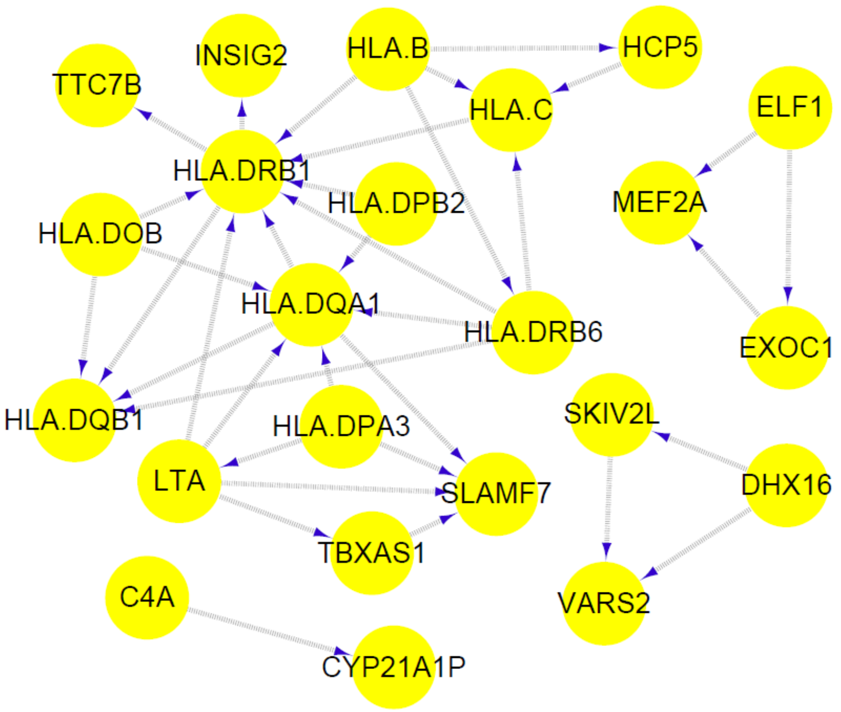

Figure S5. Co-expression network view of identified potentially pleiotropic genes for traits associated genes with WGCNA. This network includes all edges and their corresponding nodes that co-expressed with potentially pleiotropic genes with TOM ≥ 0.10.

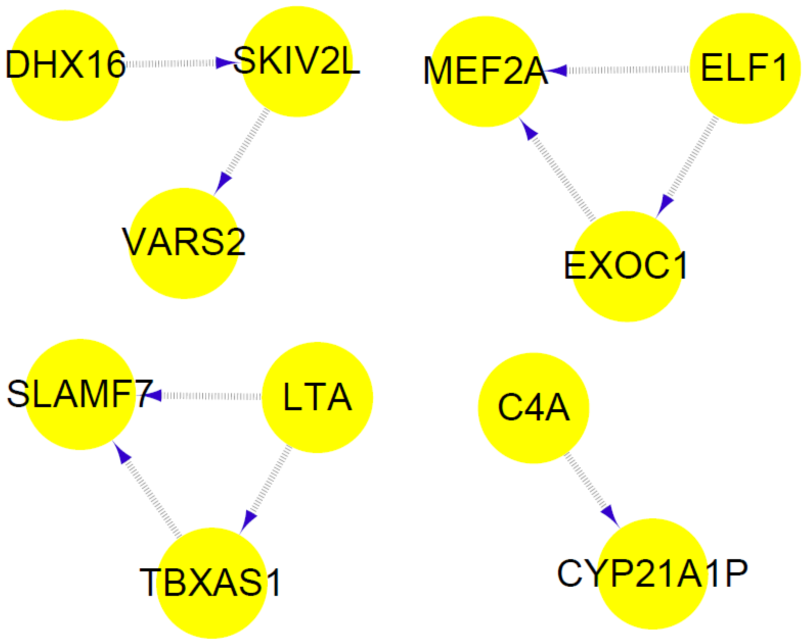

Figure S6. Co-expression network view of identified potentially pleiotropic genes for traits associated genes with GRACE. This network includes all edges and their corresponding nodes that co-expressed with potentially pleiotropic genes with TOM ≥ 0.10.

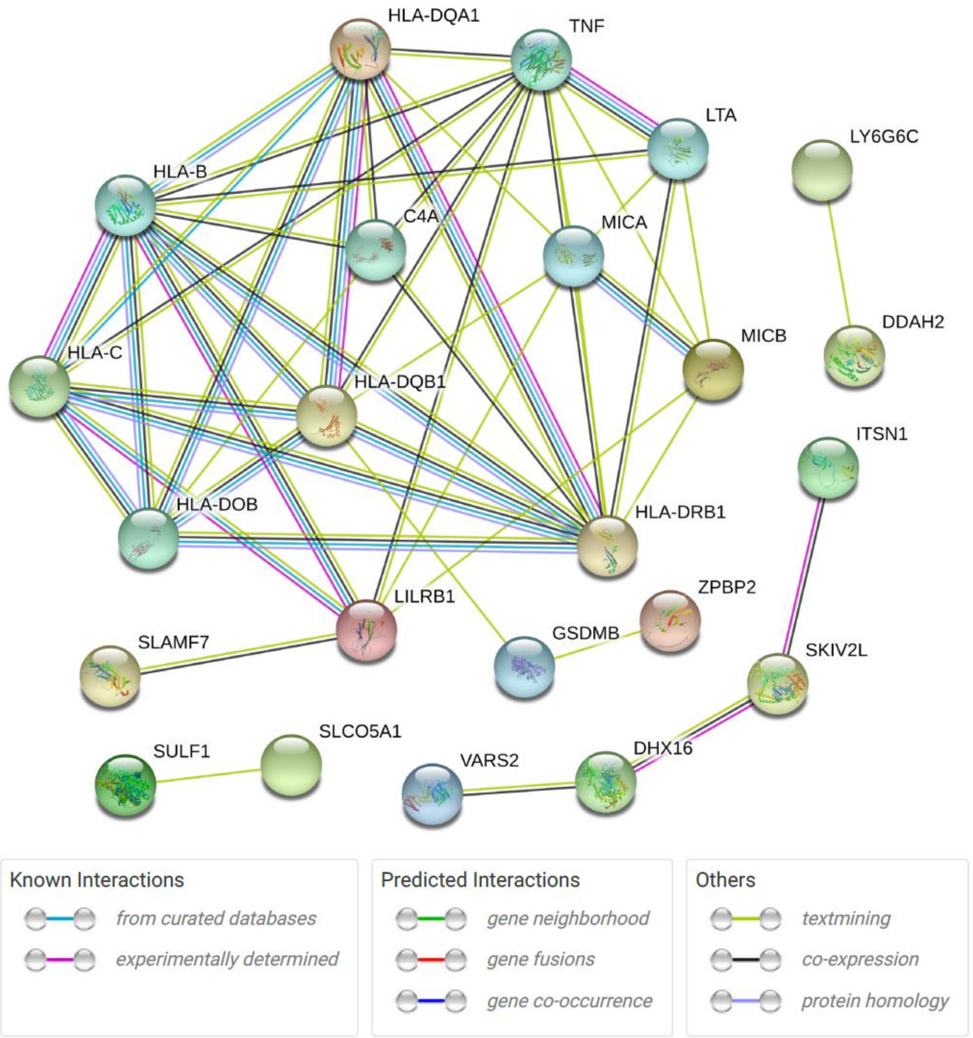

Figure S7. Protein-protein interaction analysis for both the TWAS-identified and GWAS-identified genes (the disconnected nodes in the network were hidden). Connections are based on evidence with a STRING 11.0 summary score above 0.4. Network nodes represent proteins produced by the corresponding genes, edges between nodes indicate protein-protein associations, edge color indicates the type of interaction.

References

1. Hao X, Zeng P, Zhang S, Zhou X. Identifying and exploiting trait-relevant tissues with multiple functional annotations in genome-wide association studies. PLoS genetics. 2018; 14: e1007186.
2. Milde-Langosch K, Riethdorf S. Role of cell-cycle regulatory proteins in gynecological cancer. Journal of cellular physiology. 2003; 196: 224-44.
3. Spandidos DA, Dokianakis DN, Kallergi G, Aggelakis E. Molecular basis of gynecological cancer. Annals of the New York Academy of Sciences. 2000; 900: 56-64.
4. Seeber LM, Horree N, Vooijs MA, Heintz AP, van der Wall E, Verheijen RH, et al. The role of hypoxia inducible factor-1alpha in gynecological cancer. Critical reviews in oncology/hematology. 2011; 78: 173-84.
5. Schropfer A, Kammerer U, Kapp M, Dietl J, Feix S, Anacker J. Expression pattern of matrix metalloproteinases in human gynecological cancer cell lines. BMC cancer. 2010; 10: 553.
6. Chung SH, Franceschi S, Lambert PF. Estrogen and ERalpha: culprits in cervical cancer? Trends in endocrinology and metabolism: TEM. 2010; 21: 504-11.
7. Crosbie EJ, Einstein MH, Franceschi S, Kitchener HC. Human papillomavirus and cervical cancer. Lancet (London, England). 2013; 382: 889-99.
8. Finucane HK, Bulik-Sullivan B, Gusev A, Trynka G, Reshef Y, Loh PR, et al. Partitioning heritability by functional annotation using genome-wide association summary statistics. Nature genetics. 2015; 47: 1228-35.
9. Gusev A, Ko A, Shi H, Bhatia G, Chung W, Penninx BW, et al. Integrative approaches for large-scale transcriptome-wide association studies. Nature genetics. 2016; 48: 245-52.
10. Consortium. GP, Auton A, Brooks LD, Durbin RM, Garrison EP, Kang HM, et al. A global reference for human genetic variation. Nature. 2015; 526: 68-74.
11. Barbeira AN, Dickinson SP, Bonazzola R, Zheng J, Wheeler HE, Torres JM, et al. Exploring the phenotypic consequences of tissue specific gene expression variation inferred from GWAS summary statistics. Nature communications. 2018; 9: 1825.
12. Chen D, Juko-Pecirep I, Hammer J, Ivansson E, Enroth S, Gustavsson I, et al. Genome-wide association study of susceptibility loci for cervical cancer. Journal of the National Cancer Institute. 2013; 105: 624-33.
13. Shi Y, Li L, Hu Z, Li S, Wang S, Liu J, et al. A genome-wide association study identifies two new cervical cancer susceptibility loci at 4q12 and 17q12. Nature genetics. 2013; 45: 918-22.
14. Miura K, Mishima H, Kinoshita A, Hayashida C, Abe S, Tokunaga K, et al. Genome-wide association study of HPV-associated cervical cancer in Japanese women. Journal of medical virology. 2014; 86: 1153-8.
15. Pattaro C, Teumer A, Gorski M, Chu AY, Li M, Mijatovic V, et al. Genetic associations at 53 loci highlight cell types and biological pathways relevant for kidney function. Nature communications. 2016; 7: 10023.

16. Li X, Huang K, Zhang Q, Zhou J, Sun H, Tang F, et al. Genome-wide association study identifies four SNPs associated with response to platinum-based neoadjuvant chemotherapy for cervical cancer. Scientific reports. 2017; 7: 41103.
17. Leo PJ, Madeleine MM, Wang S, Schwartz SM, Newell F, Pettersson-Kymmer U, et al. Defining the genetic susceptibility to cervical neoplasia-A genome-wide association study. Plos Genet. 2017; 13.
18. Takeuchi F, Kukimoto I, Li ZQ, Li S, Li N, Hu ZB, et al. Genome-wide association study of cervical cancer suggests a role for ARRDC3 gene in human papillomavirus infection. Hum Mol Genet. 2019; 28: 341-8.
